# Use of Wearable Technology for Measuring and Characterizing Sedentary Behavior in People With Mild Cognitive Impairment and Dementia: Systematic Review

**DOI:** 10.2196/85361

**Published:** 2026-06-25

**Authors:** Jenny L Wales, Chloe Hinchliffe, Ryan Stanley Falck, Silvia Del Din, Alison J Yarnall, Ríona Mc Ardle

**Affiliations:** 1 Faculty of Medical Sciences Translational and Clinical Research Institute Newcastle University Newcastle upon Tyne United Kingdom; 2 NIHR Newcastle Biomedical Research Centre Newcastle upon Tyne United Kingdom; 3 School of Biomedical Engineering, Djavad Mowafaghian Centre for Brain Health Faculty of Medicine University of British Columbia Vancouver, BC Canada

**Keywords:** cognitive impairment, dementia, mild cognitive impairment, sedentary behavior, systematic review, wearable technology

## Abstract

**Background:**

Sedentary behavior (SB) is a critical, modifiable risk factor for adverse health outcomes. Evidence suggests that SB is higher among individuals with cognitive impairment relative to their cognitively healthy peers. However, the nature and extent of SB across cognitive impairments remains unclear, largely due to the reliance on self-report data and the lack of synthesized evidence from more accurate methodology, such as wearable devices. Wearable device–based methodologies offer a reliable means of capturing SB in real-world settings, circumventing the recall bias inherent to self-report methods. Continuous remote monitoring of SB, via wearable devices, may provide nuanced insights important for understanding SB’s contribution to cognitive impairment and health consequences.

**Objective:**

This review aims to synthesize evidence on the volume, patterns, and variability of SB across cognitive impairment and critically appraise the wearable device–based methodology used to capture SB in this population.

**Methods:**

Following PRISMA (Preferred Reporting Items for Systematic Reviews and Meta-Analyses) guidelines, we searched 5 databases (Embase, MEDLINE, PsycInfo, Scopus, and Web of Science) up to January 2025 for peer-reviewed English-language studies using wearable devices to measure SB in community-dwelling or aged residential care residents aged 50 years or older with cognitive impairment (PROSPERO: CRD42024616523). Study quality was assessed using an adapted version of the National Institute of Health Quality Assessment Tool for Observational Cohort and Cross-Sectional Studies. Data were extracted on SB outcomes (eg, volume, pattern, and variability) and methodological characteristics (eg, device type, placement, SB classification/processing, and its corresponding validation).

**Results:**

From 2824 screened records, 17 studies (2016-2025) were included. Most studies (n=11, 65%) were of “good” quality (scoring ≥5 on bias assessment). Synthesis revealed inconsistent evidence for differences in SB volume across cognitive impairment. However, individuals with dementia consistently exhibited a unique SB pattern, engaging in significantly fewer but longer sedentary bouts than other forms of cognitive impairment and cognitively intact controls. All (n=17, 100%) studies used volume metrics to describe SB, followed by pattern metrics (n=7, 41%); only 1 study reported on SB variability. Methodological appraisal found significant heterogeneity: 13 different device models across 6 body placements were used. Most studies quantified SB using count-based thresholds (counts per minute), which were largely unvalidated in cognitively impaired or older adult populations.

**Conclusions:**

This review found that participants with dementia consistently exhibited a unique pattern of SB compared to other forms of cognitive impairment and healthy controls, while evidence for differences in SB volume was inconsistent. This may indicate that differences in SB volume are not inherent to dementia pathology but may be mediated by other factors, such as neuropsychiatric symptoms or environmental influences. Furthermore, methodological heterogeneity and unvalidated thresholds were observed throughout the review, highlighting a need for standardized protocols to enhance the validity and clinical applicability of future research.

**Trial Registration:**

PROSPERO CRD42024616523; https://www.crd.york.ac.uk/PROSPERO/view/CRD42024616523

## Introduction

Dementia is a leading global cause of disability and dependency, affecting 55 million people worldwide, a figure projected to double by 2050 [1,2]. Dementia is an umbrella term for multiple progressive neurological diseases that impact cognitive function, leading to significant impairments in functional abilities [2]. However, subtle changes in cognition often occur years before dementia diagnosis. Mild cognitive impairment (MCI) is a transition state between healthy cognitive aging and dementia, wherein cognitive performance is below the expected level for age and education level but does not interfere with independence [3,4]. Approximately 5%-15% of individuals with MCI progress to dementia each year, making it a significant risk factor [5]. Identifying modifiable lifestyle behaviors across cognitive impairment (including MCI and dementia) is therefore a critical public health priority, essential for prolonging cognitive health and reducing escalating health care expenditures [6-9].

Sedentary behavior (SB) is recognized as a distinct, modifiable risk factor for cognitive decline [7,10-12]. Although lifestyle interventions have historically targeted physical activity (PA) to promote healthy cognitive aging [13,14], emerging evidence clarifies that PA and SB are not merely opposite ends of a single continuum: individuals may meet PA guidelines yet still accumulate prolonged sedentary time that exacerbates cognitive decline [12,15-17]. This distinction carries particular significance for people with cognitive impairment (ie, MCI or dementia), who often face barriers such as executive dysfunction, apathy, and caregiver dependence that hinder active lifestyles and promote greater engagement in SB [18-21].

SB is defined as any sitting, reclining, or lying behavior, while awake, characterized by an energy expenditure ≤1.5 metabolic equivalent tasks (METs) [22-24], or any “nonupright” activity [24]. A growing body of evidence indicates that people with cognitive impairment engage in significantly more SB compared with their cognitively healthy counterparts [25]. This is quantitatively demonstrated by studies reporting that those with cognitive impairment spend up to 80% (9.7 hours) of their day sedentary [26-28], in contrast to 55% (7.7 hours) reported in the general older adult literature [29]. These quantitative findings are corroborated by qualitative evidence where 1 study found nursing home residents with cognitive impairment spend a large proportion of their day “doing nothing,” resulting in boredom, frustration, and hopelessness [30]. Collectively, these patterns underscore a critical target for intervention. First, excessive sedentary time is independently linked to exacerbated cognitive decline [15,25,27]. Second, the high volume of SB can worsen psychological symptoms commonly experienced in this population, including apathy, depression, and anxiety, thereby further diminishing overall quality of life [31-33]. This can also create an indirect pathway whereby increased SB exacerbates psychological symptoms, which in turn reduce PA engagement, forming a self-reinforcing cycle of SB [21]. Although differences in PA have been observed across cognitive impairment subtypes (eg, MCI and dementia) and severity levels (eg, mild, moderate, and severe) [34,35], it remains unclear whether SB varies similarly. The current evidence base is limited and methodologically heterogeneous [36,37]. Consequently, the precise nature and extent of SB across cognitive impairments remains inconclusive.

Accurately measuring SB is a critical first step toward developing interventions for individuals with cognitive impairment to reduce the associated health risks. The primary assessment methods offer a trade-off between context and precision. Self-report tools (eg, questionnaires) are popular for their practicality and ability to identify activity types but are undermined by recall bias and inaccuracy, which may be exacerbated by cognitive impairment [38-41]. In contrast, device-based measures are often regarded as the “gold standard” for assessing SB volume and pattern [40,42] and are recommended for assessing SB in people with cognitive impairment in free-living environments [40,41,43]. Unlike self-report tools, device-based methods enable detailed analyses of sedentary patterns, making them particularly valuable for detecting subtle behavioral changes over time and well-suited for research aiming to understand the nuanced relationship between SB and cognitive impairment [44,45]. As such, they offer a promising avenue to accurately capture SB in real-world settings with minimal participant burden [37].

Device-based methods can encompass a wide range of technologies, from smart-home systems (eg, indoor positioning systems [46]) to wearable devices (eg, accelerometers [47]). Smart-home technology can capture accurate contextual SB data in an unobtrusive manner [48]; however, it is often constrained to the home environment and requires complex infrastructure and data processing [46,48]. In contrast, wearable devices can capture SB data with high precision at a reduced cost [49]. Crucially, their ability to measure SB in free-living environments, beyond the home, provides the ecological validity necessary to understand SB patterns across all daily contexts in people with cognitive impairment [50].

Despite their advantages, wearable-based methodologies are marked by considerable heterogeneity in the SB literature [15,51]. Wearable devices primarily assess SB through 2 approaches: by quantifying a lack of movement using accelerometer cut points or detecting body posture [52]. Although posture-based measurement is considered the most accurate method for identifying SB [22,43,52,53], much of the existing literature relies on cut-point thresholds (counts per minute [CPM]). These thresholds vary substantially across studies and can produce markedly different SB estimates [54]. Beyond threshold selection, a growing body of evidence indicates that SB estimates are also influenced by broader methodological factors, including device brand (eg, activPAL and Axivity), body placement (eg, thigh or wrist), and the metrics used to characterize SB (eg, sedentary time and bout length) [44,55-60]. Such methodological inconsistencies and lack of standardization may bias estimates of SB and complicate comparisons across studies. However, whether these methodological variations influence SB estimates in populations with cognitive impairment is yet to be synthesized.

This systematic review aims to synthesize the existing evidence on SB volume, patterns, and variability (fluctuation of SB) and to critically appraise the wearable technology methods and metrics used. By identifying and analyzing the key sources of methodological variation, this work seeks to promote more standardized and appropriate measurement approaches. Addressing these methodological challenges is a critical prerequisite for advancing SB research and translating findings into practical health benefits for populations with cognitive impairment.

## Methods

### Search Strategy

This review was preregistered on PROSPERO (CRD42024616523) and designed in accordance with the PRISMA (Preferred Reporting Items for Systematic Reviews and Meta-Analyses) [61]. PRISMA 2020 checklist is available in Multimedia Appendix 1. Five databases were used for this search: Embase, MEDLINE, PsycInfo, Scopus, and Web of Science. The search criteria were restricted to studies conducted up to the date of the most recent search (January 2025), with no lower date limit. Keywords relevant to cognitive impairment, wearable digital technology, and SB formed a search string (Multimedia Appendix 2).

### Selection Criteria

Articles were screened in accordance with the most generally adopted definitions of SB: any sitting, reclining, or lying behavior, while awake, characterized by an energy expenditure ≤1.5 METs [22-24] or any nonupright activity [24].

### Inclusion and Exclusion Criteria

Articles were included if they were (1) peer reviewed; (2) published in the English language before 2025; (3) conducted within community-dwelling or aged residential care settings, including supportive living, assisted living, residential aged care, nursing homes, and care homes; (4) investigated individuals with MCI or dementia, inclusive of all subtypes to capture the full breadth of cognitive impairment presentations [62-65]; (5) involved participant samples with a mean age of ≥50 years to ensure the inclusion of MCI and young-onset dementia [62,63,66,67]; (6) reported SB metrics measured through quantitative wearable devices (eg, accelerometry); and (7) defined SB either in accordance with the established consensus definitions or through an alternative but clearly articulated and appropriate definition.

Articles were excluded if they were (1) published in a language other than English; (2) conducted in acute, in-patient hospital, or palliative care settings (as inactivity in these environments is often medically mandated and does not represent an individual’s usual, volitional behavior [68,69]); (3) focused solely on PA or sleep-related metrics; (4) assessed SB through subjective self-report measures without the inclusion of quantitative wearable-derived data; (5) involved children, adolescents, or adults without cognitive impairments, including those with subjective cognitive impairment; (6) included mixed populations without disaggregated SB data for individuals with cognitive impairment; or (7) were not primary research articles such as conference abstracts, conference proceedings, posters, study protocols, letters to the editor, reviews, meta-analyses, or gray literature.

### Data Extraction

All titles, abstracts, and full texts were independently screened by 2 reviewers (JLW and CH) using Rayyan software developed by Ouzzani et al [70]. Any disagreements were settled by a third reviewer (RMA).

Author JLW created data extraction forms using Excel (Microsoft Corp) and refined them in collaboration with 2 other authors (CH and RMA). One author (JLW) independently extracted data from all eligible articles, and the extracted data were verified by author CH.

The key measures of interest were: (1) study design, (2) participant characteristics and study setting, (3) stage of cognitive impairment (ie, MCI and mild, moderate, or severe dementia) and diagnostic criteria applied, (4) measure of outcome (SB), and (5) comparisons between groups (healthy controls or other forms of cognitive impairment). For outcome measures (SB), we extracted the (1) SB metrics and their values (eg, volume, pattern, and variability metrics); (2) method of assessment (eg, accelerometry); (3) definition of measurement (eg, body posture or cut-point thresholds); (4) device name and manufacturer; (5) data collection procedure (eg, device placement and length of assessment); (6) previously established validity and reliability of SB classification (eg, validation of cut point); and (7) data processing algorithms used to derive SB outcomes, including the classification approach (eg, cut-point–based or posture-based), algorithm source (eg, academic or proprietary), and evidence of algorithm validation.

### Assessment of Study Quality

The quality of study articles was assessed using an adapted version of the National Institute of Health Quality Assessment Tool for Observational Cohort and Cross-sectional Studies [71]. This adapted version has previously been used in similar reviews [72]. Study quality was assessed based on 7 questions, which can be found in Multimedia Appendix 3.

Two raters (JLW and CH) independently assessed the quality of the studies and reached consensus through discussion; average scores determined the overall quality of each study. A binary system was used to rate the studies (1=yes and 0=no), which enabled a summed score out of 7 to aid in determining the overall quality of each study (rated good: scores 5-7, moderate: scores 3-4, or poor: scores 0-2).

### Data Synthesis

Narrative data synthesis was applied to summarize and integrate findings across the studies. Reflecting this systematic review’s aims, results were grouped by (1) synthesis of reported SB outcomes in people with cognitive impairment, considering disease severity and subtype; and (2) wearable technology methods and metrics used to measure SB, including device type and placement, characterization of SB, and processing techniques and validity. Due to heterogeneity in the data, meta-analysis was infeasible.

To support the interpretation of data and provide consistency across the literature, we categorized wearable technology metrics of SB into volumes, patterns, and variability of behavior. These categories were theoretically derived, drawing on established concepts in SB and PA literature [24,72-74], and were operationalized before the final data synthesis to ensure consistent classification of metrics across all included studies. Volume refers to the amount of time spent in SB during a specified period (eg, per day or full assessment period), expressed either as an absolute value (minutes or hours) or as a relative value (percentage of time) [22]. Where necessary, time-based data were converted to a common unit (eg, minutes) to ensure consistency in aggregation and comparison. Pattern explains the way sedentary time is accumulated, including the number and duration of SB bouts, the interruptions in SB, and the distribution of SB throughout the day [24]. Variability refers to the intraindividual fluctuations or day-to-day changes in sedentary time and captures how consistently (or inconsistently) a person engages in SB across different days or within various segments of the day (eg, morning vs evening) [75]. The categorization of metrics was performed by the primary researcher (JLW) and reviewed by all authors. Any amendments were discussed collaboratively, and all revisions were agreed upon by consensus.

## Results

### Search Yield

**Figure 1:**
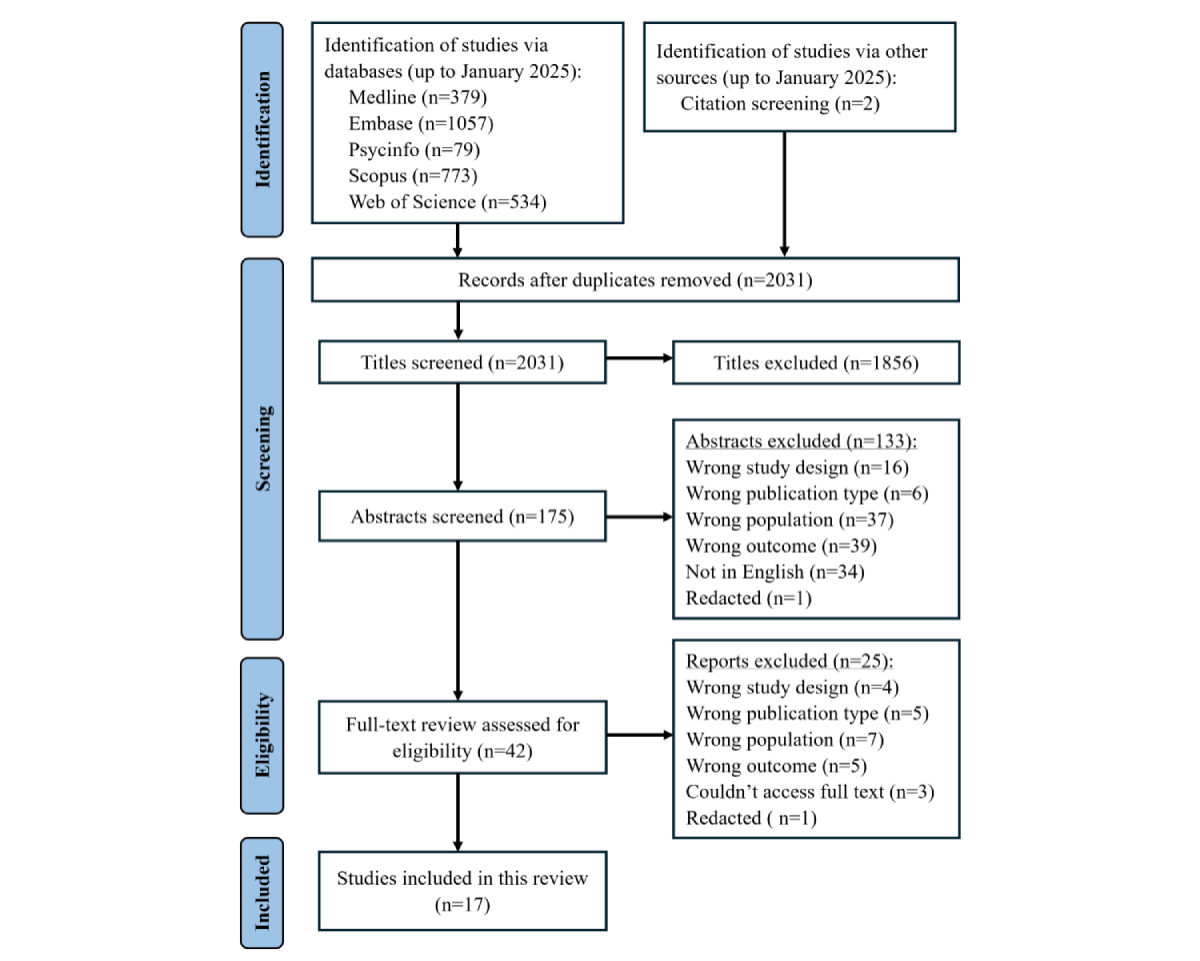
PRISMA (Preferred Reporting Items for Systematic Reviews and Meta-Analyses) flow diagram demonstrating the search yield for this systematic review.

Figure 1 describes the results of the search strategy for articles investigating SB using wearable digital technology in people with cognitive impairment. The search was conducted between November 2024 and January 2025 and initially yielded 2822 articles; 2 additional studies were identified through citation screening. Our final systematic review included 17 articles, all of which were published between 2016 and 2025.

### Study Characteristics

**Figure 2:**
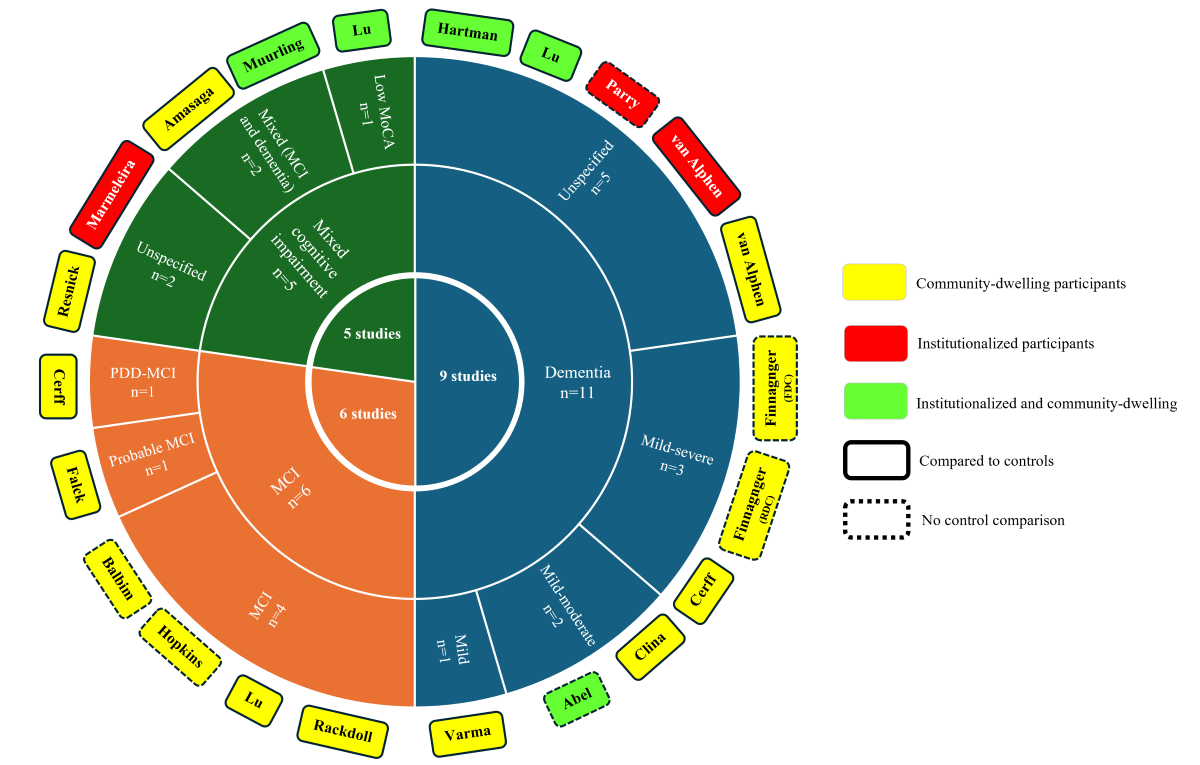
Overview of cognitive impairment categorization, study population, and control group comparisons for all studies included in this review. FDC: farm-based dementia care; MCI: mild cognitive impairment; MoCA: Montreal Cognitive Assessment; PDD: Parkinson disease dementia; RDC: regular day care.

Table 1 contains information relating to all the study characteristics. Studies took place in Germany (3/17, 18%), the Netherlands (3/17, 18%), the United States of America (3/17, 18%), Australia (2/17, 12%), Canada (2/17, 12%), Hong Kong (1/17, 6%), Japan (1/17, 6%), Norway (1/17, 6%), and Portugal (1/17, 6%). The sample size of participants with cognitive impairment ranged between 8 and 539 across all studies, with a mean age range of 68-91.7 years. Regarding participants with cognitive impairment, 59% (10/17) of studies reported ≥50% of participants as female. A total of 3 (18%) studies reported the ethnicity of participants with cognitive impairment, whereby more than 87% of the participants were White. Most studies were cross-sectional (14/17, 82%), the remaining studies (3/17, 18%) used baseline data from randomized controlled trials or intervention studies; there were no longitudinal observational studies. Of the 17 studies, 11 included only community-dwelling participants, 3 included only institutionalized participants, and 3 included a mix of both (Figure 2 [76-92]).

**Table 1:** Summary of included studies with main findings for SB^a^ metrics reported in ≥2 studies.

Study; quality	Participant demographics	Data collection and processing	Main study findings	Comparisons
Abel et al [76]; good	Dementia: n=53 (age: 82.3 years; 74% female; 72% community-dwelling)	Device: PAMSys (chest) Duration: 3 days Classification: body posture Algorithm: academic	Lying: 9.1 hours/day Sitting: 11.3 hours/day Inactive: 20.4 hours/day	No comparisons
Amagasa et al [77]; moderate	Cognitive impairment: n=48 (MCI^b^/AD^c^; age: 77.6 years; 52% female; 100% community-dwelling) Healthy controls: n=463 (age: 73, SD 5.4 years; 53% female; 100% community-dwelling)	Device: HJA-750C (waist) Duration: 7 days Classification: ≤1.5 METs^d^ Algorithm: academic	SB time: cognitive impairment, 476.2 minutes/day; healthy controls, 442.4 minutes/day	SB time was not significant vs healthy controls
Balbim et al [78]; good	MCI: n=253 (age: 73.69 years; 62% female; 100% community-dwelling)	Device: Motionwatch8 (wrist) Duration: 7 days Classification: ≤178.5 CPM^e^ Algorithm: proprietary	SB time: 626.9 minutes/day SB percentage: 43.55% of the day	No comparisons
Cerff et al [79]; good	PDD^f^-MCI: n=22 (age: 68 years; 23% female; 100% community-dwelling) PDD: n=9 (72 years; 0% female; 100% community-dwelling) Healthy controls: n=17 (age: 71 years; 41% female; 100% community-dwelling)	Device: MiniMod (lower back) Duration: 3 days Classification: body posture Algorithm: proprietary	SB percentage: PD-MCI, 78% of the day; PDD, 89% of the day; healthy controls, 75% of the day SB bouts: PD-MCI, 129/day; PDD, 97/day; healthy controls: 134/day SB bout length: PD-MCI, 515 seconds; PDD, 727 seconds; healthy controls, 506 seconds	PDD: ↓ bouts and ↑ bout length vs PD-MCI and healthy controls. SB percentage was not significant
Clina et al [80]; good	AD: n=65 (age: 73.6 years; 37% female; 100% community-dwelling) Healthy controls: n=65 (age: 69.4 years; 69% female; 100% community-dwelling)	Device: wGT3x-BT (hip) Duration: 7 days Classification: ≤100 CPM Algorithm: custom	SB time: AD, 622.2 minutes/day; healthy controls: 591.8 minutes/day	SB time was not significant vs healthy controls
Falck et al [81]; good	MCI: n=81 (age: 72.5 years; 59% female; 100% community-dwelling) Healthy controls: n=69 (age: 69.4 years; 78% female; 100% community-dwelling)	Device: Motionwatch8 (wrist) Duration: ≥4 days Classification: ≤178.5 CPM Algorithm: proprietary	SB percentage: MCI, 61.65% of the day; healthy controls, 57.24% of the day SB bouts (≥30 minutes): MCI, 4.07/day; healthy controls, 3.30/day	MCI: ↑bouts ≥30 minutes vs Healthy controls. SB percentage was not significant
Finnanger et al [82]; moderate	Dementia: n=29 (FDC^g^; age: 74 years; 31% female; 100% community-dwelling) Dementia: n=107 (RDC^h^; age: 84.3 years; 66% female; 100% community-dwelling)	Device: Actisleep+ (wrist) Duration: 7 days Classification: ≤99 CPM Algorithm: proprietary	SB percentage: FDC, 39.7% of the week; RDC, 43.51% of the week	Findings between care settings were not significant
Hartman et al [83]; good	Dementia: n=45 (age: 79.6 years; 49% female; 93% community-dwelling) Healthy controls: n=49 (age: 80 years; 51% female; 98% community-dwelling)	Device: Actiwatch 2 (wrist) Duration: 7 days Classification: ≤145 CPM Algorithm: proprietary	SB time: dementia, 8.5 hours/day; healthy controls, 8.2 hours/day SB percentage: dementia, 57% of the day; healthy controls, 55% of the day SB bouts (≥30 minutes): dementia, 2.3/day; healthy controls, 2.0/day SB bout length: dementia, 18.3 minutes; healthy controls, 16.6 minutes	Dementia: ↑ SB percentage and ↑ bout length vs healthy controls. SB time and bouts ≥30 minutes was not significant
Hopkins et al [84]; good	MCI: n=82 (age: 72 years; 56% female; 100% community-dwelling)	Device: ActivPAL (thigh) Duration: 7 days Classification: body posture Algorithm: proprietary	SB time: 637 minutes/day Sitting: 558 minutes/day Lying: 21 minutes/day SB bouts ≥30 minutes: 5/day	No comparisons
Lu et al [85]; moderate	MCI: n=105 (age: 83.6 years; 49% female; 100% community-dwelling) Low MoCA^i^: n=252 (age: 83.4 years; 48% female; 98% community-dwelling) AD: n=182 (age: 80.8 years; 66% female; 98% community-dwelling) Healthy controls: n=271 (age: 81.9 years; 38% female; 100% community-dwelling)	Device: wGT3x-BT (wrist) Duration: 7 days Classification: <1853 VM^j^ CPM Algorithm: proprietary	SB percentage: MCI, 57.1% of the day; Low MoCA, 57.8% of the day; AD, 63.2% of the day; healthy controls, 58.4% of the day SB bout length: MCI, 6.3 minutes; Low MoCA, 6.5 minutes; AD, 7.9 minutes; healthy controls, 6.6 minutes SB bouts: MCI, 89.4/day; Low MoCA, 91.4/day; AD, 86.1/day; healthy controls, 91.5/day SB bouts ≥30 minutes: MCI, 3.5/day; Low MoCA, 3.3/day; AD, 4.1/day; healthy controls 3.3/day	AD: ↑ SB percentage, ↑ bout length, ↑ ≥30-minute bouts vs MCI, Low MoCA, healthy controls, and ↓ bouts vs Low MoCA and healthy controls
Marmeleira et al [86]; good	Cognitive impairment: n=48 (age: 83.9 years; 73% female; 0% community-dwelling) Healthy controls: n=22 (age: 82.2 years; 55% female; 0% community-dwelling)	Device: GT1M (hip) Duration: 7 days Classification: ≤100 CPM Algorithm: proprietary	SB time: cognitive impairment, 603.7 minutes/day; healthy controls, 601.0 minutes/day SB percentage: cognitive impairment, 87.2% of the day; healthy controls, 84% of the day	All findings were not significant vs healthy controls
Muurling et al [87]; good	Cognitive impairment: n=12 (MCI/AD; age: 91.7 years; 67% female; 83% community-dwelling) Healthy controls: n=24 (age: 92.5 years; 45% female; 94% community-dwelling)	Device: MoveMonitor (lower back) Duration: 7 days Classification: body posture Algorithm: proprietary	SB time: cognitive impairment, 142.7 hours/week; healthy controls, 140.7 hours/week Sitting bouts: cognitive impairment, 681.5/week; healthy controls, 741.1/week	All findings were not significant vs healthy controls
Parry et al [88]; moderate	Dementia: n=8; healthy controls: n=20 (age: 83.1 years; 29% female; 0% community-dwelling)	Device: GT3X (dementia, hip; healthy controls, thigh) Duration: 5 days Classification: ≤100 CPM Algorithm: custom	SB time: dementia, 565.6 minutes/day; healthy controls, 583.9 minutes/day SB percentage: dementia, 86% of the day; healthy controls, 85% or the day	No comparisons
Rackdoll et al [89]; moderate	MCI: n=18 (age: 70 years; 56% female; 100% community-dwelling) Healthy controls: n=48 (age: 65 years; 52% female; 100% community-dwelling)	Device: GT3X+ (hip) Duration: 7 days Classification: ≤99 CPM Algorithm: proprietary	SB percentage: MCI, 72% of the day; healthy controls 74% of the day	SB percentage was not significant vs healthy controls
Resnick et al [90]; good	Cognitive impairment: n=279 CI; healthy controls: n=101 (age: 89.5 years; 72% female; 0% community-dwelling)	Device: Motionwatch8 (wrist) Duration: 5 days Classification: ≤178.5 CPM Algorithm: proprietary	SB time: cognitive impairment, 1203 minutes/day; healthy controls, 1138 minutes/day	Cognitive impairment ↑ SB time vs healthy controls
van Alphen et al [91]; good	Dementia (community-dwelling): n=37 (age: 77.3 years; 41% female; 100% community-dwelling) Dementia (institutionalized): n=83 (age: 83 years; 80% female; 0% community-dwelling) Healthy controls: n=26 (79.5 years; 50% female; 100% community-dwelling)	Device: Actiwatch AW4 (wrist) Duration: >6 days Classification: ≤100 CPM Algorithm: not specified	SB time: dementia (institutionalized), 17.3 hours/day; dementia (community-dwelling), 15.83 hours/day; healthy controls, 14.52 hours/day	Dementia ↑ SB time vs healthy controls Dementia (institutionalized) ↑ SB time vs dementia (community-dwelling) and healthy controls
Varma et al [92]; moderate	AD: n=39 (age: 73.5 years; 28% female; 100% community-dwelling) Healthy controls: n=53 (age: 73.2 years; 70% female; 100% community-dwelling)	Device: GT3X+ (hip) Duration: 7 days Classification: ≤149 CPM Algorithm: proprietary	SB percentage: AD, 60.94% of the day; healthy controls, 54.07% of the day	SB percentage was not significant vs healthy controls

^a^SB: sedentary behavior.

^b^MCI: mild cognitive impairment.

^c^AD: Alzheimer disease.

^d^METs: metabolic equivalent tasks.

^e^CPM: counts per minute.

^f^PDD: Parkinson disease dementia.

^g^FDC: farm-based dementia care.

^h^RDC: regular day care.

^i^MoCA: Montreal Cognitive Assessment.

^j^VM: vector magnitude.

Levels of cognitive impairment described across the 17 studies included MCI (n=5, 25%), unspecified severity of dementia (n=4, 20%), unspecified level of cognitive impairment (n=3, 15%), mild-moderate dementia (n=2, 10%), mild-severe dementia (n=2, 10%), mix of MCI and dementia (n=2, 10%), probable MCI (n=1, 5%), and mild dementia (n=1, 5%) (Figure 2).

For the purposes of this review, cognitive impairment was categorized into 3 broader cohorts: MCI (n=6), dementia (n=11), and mixed cognitive impairment (n=5), spanning the 17 studies. Mixed cognitive impairment refers to cohorts that have been identified as cognitively impaired with no specific diagnosis or mixed groups of dementia and MCI. Overall, 12 studies (71%) included comparisons to controls, resulting in 16 control cohorts corresponding to the dementia (n=7), MCI (n=4), and mixed cognitive impairment (n=5) cohorts (Figure 2).

A total of 8 (47%) studies specified dementia disease subtypes, of which 88% (n=7) included participants with Alzheimer disease (AD) [77,80,85,87,91-93]; 25% (n=2) reported participants with MCI/dementia due to Parkinson disease [79,91]; 25% (n=2) involved participants with vascular dementia or mixed dementia [91,93]; and 13% (n=1) included participants with frontotemporal dementia, dementia with Lewy bodies, or Korsakoff syndrome [91]. Additionally, 15 (88%) studies explicitly described procedures to characterize cognitive impairment (eg, clinician review and cognitive score thresholds), and 80% (n=12) of these used validated diagnostic criteria.

### Quality Assessment

Mostly, studies were of adequate quality, with 65% (11/17) being rated as “good” and 35% (6/17) being rated as “moderate.” No studies were rated “poor.” The most common reasons for reduced study quality were a lack of sample size justification (n=15) (Multimedia Appendix 3).

### Aim 1: Volume Patterns and Variability of SB in People With Cognitive Impairment

#### Overview

A summary of extracted outcomes (metrics reported by ≥2 studies) is provided in Table 1, with all metric definitions and prevalence detailed in Table 2. A comprehensive narrative synthesis of all extracted outcomes is provided in Multimedia Appendix 4.

**Table 2:** Prevalence and descriptions of SB^a^ metrics captured in this review.

SB metric		Frequency, n (%)	Description
Volume			
	Volume of SB	17 (100)	Time spent in SB for a specified time frame (eg, per day/per week) expressed as an absolute value (hours/minutes/seconds) or relative value (percentage of time)
	SB time	10 (59)	Amount of time (hours/minutes/seconds) spent in SB over a specified period (eg, per day/week)
	SB proportion	10 (59)	Percentage (%) of time spent in SB over a specified period (eg, per day/week)
	Lying time	2 (12)	The length of time spent in sedentary lying (hours/minutes/seconds)
	Sitting time	2 (12)	The length of time spent in sedentary sitting (hours/minutes/seconds)
	Inactive time	1 (6)	The length of time spent inactive (sum of lying and sitting time) (hours/minutes/seconds)
	Lying proportion	1 (6)	Relative percentage of time spent in sedentary lying
	Sitting proportion	1 (6)	Relative percentage of time spent in sedentary sitting (%)
	SB proportion (METs^b^)	1 (6)	Relative percentage of time spent with intensity levels of ≤1.5 METs (%)
Pattern			
	Pattern of SB	7 (41)	The number of sessions and distributions of sedentary activity over a specified period (eg, per day/week)
	SB bouts (≥30 minutes)	4 (53)	The number of sedentary bouts lasting over 30 minutes, continuously
	SB bouts	3 (18)	The number of sedentary bouts accumulated over a specified time (eg, per day/week)
	Mean SB bout length	3 (18)	The average length of time spent in a singular sedentary bout
	SB interruptions	1 (6)	The count of nonsedentary bouts that occur between two sedentary bouts
	SB bouts (≥60 minutes)	1 (6)	The number of sedentary bouts lasting over 60 minutes, continuously
	Hourly SB distribution	1 (6)	The extent to which an individual’s SB differs across hours of the day
Variability			
	Variability of SB	1 (6)	Fluctuations in sedentary time or patterns across a specified time (eg, day-to-day) within individuals or groups over time
	Day-to-day variability	1 (6)	The extent to which an individual’s SB differs across multiple days

^a^SB: sedentary behavior.

^b^METs: metabolic equivalent tasks.

#### Volume

All 17 studies used volume metrics to describe SB in individuals with cognitive impairment. Nine studies (53%; 10 cohorts) reported SB time per day: 4 in dementia (5 cohorts; ranging from 510 to 1038 minutes per day) [80,88,91,93], 2 in MCI (ranging from 626.9 to 637 minutes per day) [78,84], and 3 in mixed cognitive impairment (ranging from 476.2 to 1203 minutes per day) [77,86,90]. A total of 3 out of 4 dementia studies considered control group comparisons [80,91,93]; only 1 study [91] reported significantly higher SB time in institutionalized participants with dementia per day relative to both community-dwelling participants with dementia (*P*<.05) and healthy controls (*P*<.001), while the remaining 2 dementia studies reported no significant differences in SB time per day [80,93]. In MCI, neither of the 2 studies performed comparative analyses with control groups. For mixed cognitive impairment, all 3 studies considered control comparisons; only 1 study reported significantly higher SB in those with unspecified cognitive impairment (*P*<.01) [90], with the others reporting no significant differences [77,86]. Additionally, 1 study [87] reported SB time per week in mixed cognitive impairment (142.7 hours per week) and found no significant differences when compared with controls.

Nine studies (53%; 12 cohorts) reported SB as a percentage of the day: 5 in dementia (ranging from 57% to 89% per day) [79,85,88,92,93], 5 in MCI (ranging from 43.55% to 78% per day) [78,79,81,85,89], and 2 in mixed cognitive impairment (ranging from 57.8% to 87.2% per day) [85,86]. Among dementia studies, 4 included control comparisons [79,85,92,93], 2 reported statistically higher SB proportion (*P*<.05) [85,93], while the others found no significant differences [79,92]. Contrastingly, 4 of 5 MCI studies [79,81,85,89] and both mixed cognitive impairment studies considered group comparisons with controls, reporting no statistically significant differences. Additionally, 1 study [82] reported SB as a percentage of the week in 2 dementia cohorts (ranging from 39.7% to 43.51% per week), without comparison with healthy controls.

Two studies [76,84] assessed SB as sitting and lying minutes per day, respectively, 1 in dementia [76] (sitting: 678 minutes; lying: 546 minutes) and 1 in MCI [84] (sitting: 558 minutes; lying: 21 minutes). Neither incorporated control comparisons, and no studies in mixed cognitive impairment reported this metric.

Finally, 1 study [79] assessed SB in people with dementia and MCI compared with cognitively intact controls using 3 volume metrics. First, it measured the proportion of time spent at ≤1.5 METs, finding that participants with dementia were sedentary for 89% of the day, compared with 84% for those with MCI. However, these differences were not statistically significant, either between MCI and dementia (*P*=.92) or between dementia and cognitively intact controls (*P*=.64). Second, the study reported the proportion of time spent lying and sitting. The MCI group spent the largest proportion of their day lying (42%), followed by sitting (34%), whereas the group with dementia spent more time sitting (42%) than lying (40%). However, no statistical comparisons were reported for this postural data, and these metrics were not assessed in mixed cognitive impairment groups.

#### Pattern

Seven studies (41%) used pattern metrics to describe SB in individuals with cognitive impairment. Four studies (57%; 6 cohorts) reported the number of prolonged (≥30 minutes) SB bouts per day: 2 in dementia (ranging from 2.3 to 4.1 bouts per day) [85,93], 3 in MCI (ranging from 3.5 to 5 bouts per day) [81,84,85], and 1 in mixed cognitive impairment (3.3 bouts per day) [85]. In dementia, both studies considered comparisons with controls [85,93]; 1 reported a significantly higher number of prolonged bouts compared with healthy controls, MCI, and mixed cognitive impairment groups (*P*<.05) [85], while the other found no significant differences [93]. In MCI, 2 of the 3 studies conducted comparative analyses with controls [81,85], with one reporting significantly more prolonged bouts than healthy controls (*P*<.05) [81] and the other finding no significant difference [85]. For mixed cognitive impairment, the single study found no statistically significant differences compared with controls [85].

Three studies (43%; 6 cohorts) reported average SB bout length: 3 in dementia (ranging from 474 to 1098 seconds) [79,85,93], 2 in MCI (ranging from 378 to 515 seconds) [79,85], and 1 in mixed cognitive impairment (390 seconds) [85]. All studies considered comparisons with control groups. All 3 dementia studies reported significantly longer bout durations compared with healthy controls (*P*<.05); 1 study further reported that this difference remained significant when compared to both MCI and mixed cognitive impairment groups [85]. In contrast, studies of MCI and mixed cognitive impairment found no significant differences in average bout length vs controls.

Two studies (43%; 5 cohorts) reported the number of SB bouts per day: 2 in dementia (ranging from 86.1 to 97.0 bouts per day) [79,85], 2 in MCI (ranging from 89.4 to 129.0 bouts per day) [79,85], and 1 in mixed cognitive impairment (91.4 bouts per day) [85]. All studies conducted comparative analyses with controls. For participants with dementia, both studies reported significantly fewer SB bouts compared with healthy controls (*P*<.05); 1 study further noted this finding remained significant when compared with a group with mixed cognitive impairment, but not MCI [85]. In contrast, studies of MCI and mixed cognitive impairment found no significant differences in this metric compared with controls. Additionally, 1 study reported the number of sitting bouts per week in mixed cognitive impairment (MCI and AD) [87], reporting 681.5 sitting bouts per week, with no significant differences compared with controls.

One study [93] compared daily sedentary interruptions between participants with dementia and healthy controls. Although the group with dementia averaged 27.2 interruptions per day, this difference was not statistically significant (*P*=.20). This metric was not assessed in participants with MCI or mixed cognitive impairment.

Another study [84] quantified the daily number of sedentary bouts exceeding 60 minutes in individuals with MCI, finding an average of 1 bout per day. However, this study did not consider control group comparisons, and this metric was not assessed in groups with dementia or mixed cognitive impairment.

Finally, 1 study [86] reported the hourly distribution of SB and found participants with unspecified cognitive impairment spent significantly more time sedentary, compared with healthy controls, during the hours of 7 AM to 11 AM, 1 PM, and 4 PM. This metric was not assessed in participants with dementia or MCI.

#### Variability

One study [76] reported day-to-day variability of participants with dementia, where they found SB parameters demonstrated consistently low day-to-day variability between Friday and Saturday and Saturday and Sunday (intraclass correlation coefficients ranging from 0.82 to 0.85), indicating minimal influence from day-specific or contextual factors. This metric was not assessed in participants with MCI or mixed cognitive impairment.

### Aim 2: Wearable Digital Methods and Metrics Used to Measure SB in People With Cognitive Impairment

#### Overview

**Figure 3:**
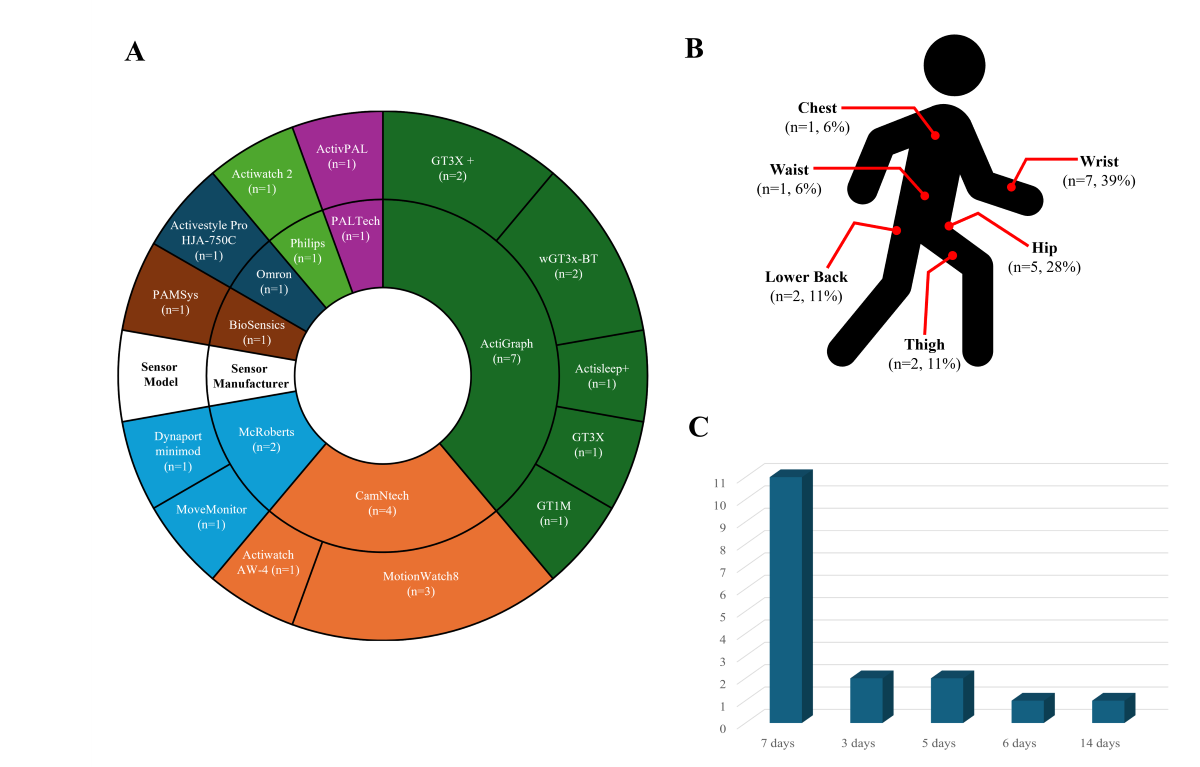
Methods and protocols used across studies included in this review. (A) Digital devices used to capture sedentary behavior (SB) metrics categorized by sensor type and manufacturer, (B) prevalence of placements of wearable devices on the body, and (C) prevalence of SB collection time periods.

All studies (100%) used accelerometer-based wearable devices to measure SB in people with cognitive impairment. Across the 17 studies, 13 different device models were used across 7 different manufacturing brands (Figure 3A). The most common manufacturing brand reported was ActiGraph, with 41% (7/17) of studies using 5 different ActiGraph models to measure SB. However, the MotionWatch8 (CamNtech Ltd) was the most used wearable device across the studies (3/17, 18%). Six different body placements were used for devices, the most common placement being the wrist (7/17, 39%) (Figure 3B). Five different assessment periods were reported by studies; most study protocols (11/17, 65%) requested participants to wear the devices for 7 days (Figure 3C).

#### Data Loss and Wear Compliance

Thirteen (76%) papers reported reasons for data loss, including insufficient data collection (n=11, 92%; <3 days [82,85,86]; <4 days [87,88]; <5 days [78]; <6 days [93]; <6 consecutive days [91]; <80% daily wear time [79]; <10 hours daily [84]; <10 hours on at least 3 days including 1 weekend day [80]), technical issues (n=6, 46% [87-91,93]), removal or refusal to wear devices (n=3, 23%; wrist [90,91]; lower-back [87]), lost devices (n=2, 17% [87,91]), forgetting to wear the device (n=1, 8% [87]), and organizational issues (n=1, 8% [91]).

#### Nonwear Identification and Wear Time Criteria

Eleven studies (65%) explicitly reported identifying nonwear in their analyses, either by specifying the criteria used (eg, zero accelerometer counts for a specified period, n=8 [77,80,81,85,86,88,90,92]) or by referencing nonwear algorithms (n=3 [79,82,87]). The remaining studies either did not report (n=4 [76,84,89,91]) or explicitly did not perform (n=2 [78,93]) nonwear identification. Fourteen studies (82%) established thresholds to determine data validity. The minimum required days for inclusion ranged from 3 to 6 days (n=11), and the minimum required wear per day ranged from 8 to 24 hours (n=11) (Multimedia Appendix 4).

#### SB Quantification and Algorithm Validation

All studies (100%) reported their quantification of SB. A total of 4 (24%) classified SB using body posture [76,79,84,87], and 1 study (6%) classified SB as ≤1.5 METs [77]. A total of 71% (12/17) used CPM thresholds to classify SB.

**Figure 4:**
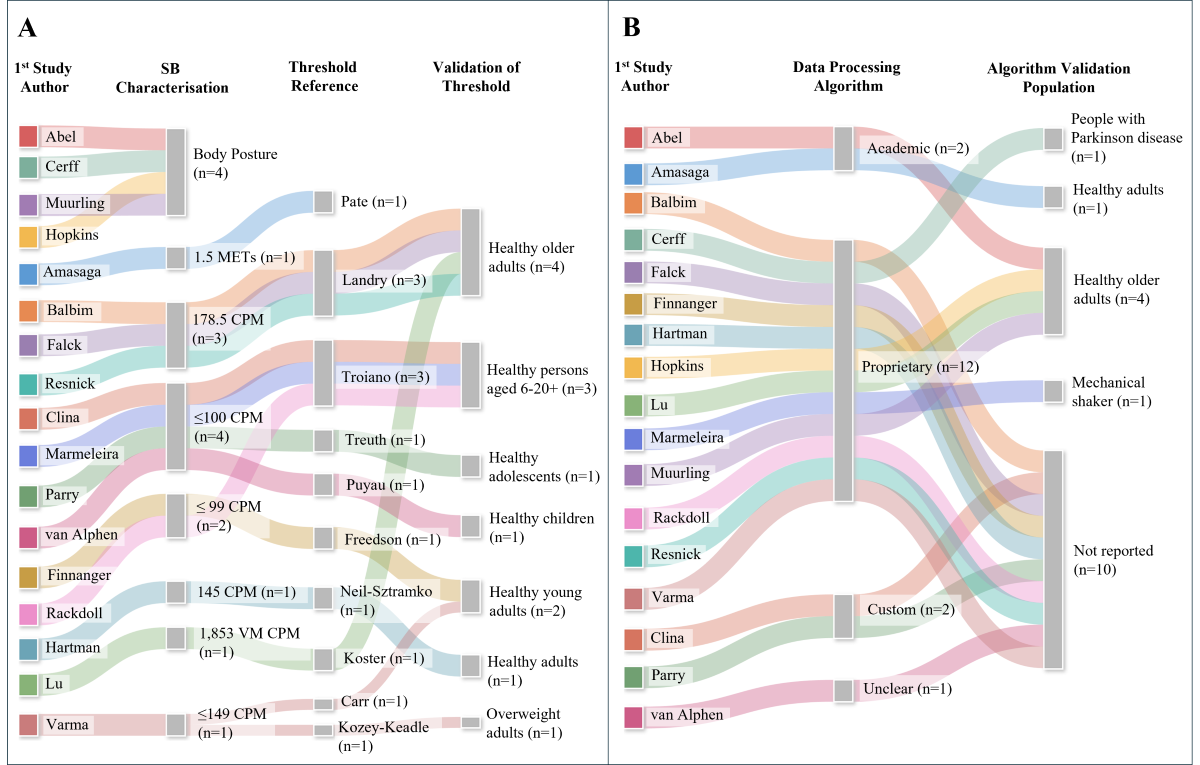
Variability in sedentary behavior (SB) characterization, thresholds, and processing across all studies (n=17). (A) Approaches to SB characterization, adopted thresholds, and validation of thresholds. (B) Data processing algorithms used to process SB with corresponding validation populations. CPM: counts per minute; METs: metabolic equivalent tasks; VM: vector magnitude.

In these cases, the thresholds were supported by references to validation studies conducted in 7 distinct populations (Figure 4A [76-92]). Furthermore, 16 (94%) studies described the data processing algorithms used to extract SB outcomes, 12 (75%) used proprietary algorithms provided by the manufacturer, 2 (13%) adopted academic algorithms provided from previous research, and 2 (13%) used custom-developed algorithms. Further, 7 (44%) studies explicitly reported the validation of the algorithms to extract SB outcomes (Figure 4B).

### Influence of Methodological Approaches on SB Estimates

The choice of classification approach appeared to influence the types of metrics reported across the included studies. Studies using posture-based classification were more likely to report pattern-related metrics (3/4, 75%) compared with those using CPM thresholds (4/12, 33%). The MET-based study did not report any pattern-related metrics. Day-to-day variability was the least frequently captured dimension, reported in only 1 study [76], which used a posture-based approach. The specific parameters used by each study are detailed in Multimedia Appendix 5, and definitions for all identified metrics are provided in Table 2. As SB volume was the only outcome reported by all studies, it was used to examine how methodological choices shaped the resulting estimates. Volume metrics reported by at least 2 studies are synthesized below and in Table 1, with a complete overview provided in Multimedia Appendix 4.

### Device Placement

All CPM-based studies (n=12) applied devices on the wrist (n=7 [78,81,82,85,90,91,93]) or hip (n=5 [80,86,88,89,92]). Wrist-worn SB time ranged from 510 to 1203 minutes per day (n=4 [78,83,90,91]), and the proportion ranged from 43.55% to 63.2% per day (n=4 [78,81,83,85]). Hip-worn SB time ranged from 565.6 to 622.2 minutes per day (n=3 [80,86,88]), and proportion ranged from 60.94% to 87.2% per day (n=4 [86,88,89,92]). Posture-based studies (n=4) applied devices on the lower back (n=2 [79,87]), thigh (n=1 [84]), or chest (n=1 [76]). Lower‑back placement produced sedentary proportions of 78% to 89% per day [79]. Thigh placement reported 637 minutes of SB time per day [84], while chest reported 1224 minutes per day [76]. The single MET-based study (n=1 [77]) used a waist-mounted device and reported 476.2 minutes of SB time per day.

### Classification Approach

Among the studies adopting the lowest cut-point thresholds of ≤99-100 CPM, SB time per day ranged from 565.6 to 1038 minutes (n=4 [80,86,88,91]), while SB proportion per day ranged from 72% to 87.2% (n=3 [86,88,89]). In studies adopting mid-range thresholds of ≤145-149 CPM, SB proportion ranged from 57% to 60.94% (n=2 [92,93]). For those using a higher threshold of ≤178.5 CPM, SB time per day ranged from 626.9 to 1203 minutes (n=2 [78,90]), with SB proportion per day ranging between 43.55% and 61.65% (n=2 [78,81]).

Posture-based studies generally provided more granular data than threshold-based methods. Two such studies reported daily SB time (637-1224 minutes per day) and further distinguished between sitting (558-678 minutes per day) and lying time (21-546 minutes per day) [76,84]. Additionally, a study using body-posture classification estimated the proportion of SB spent at ≤1.5 METs to be between 84% and 89% per day [79]. The single MET-based study estimated SB time ≤1.5 METs (476.2 minutes per day) [77].

### Denominator Choice in Proportion Calculations

Of the 10 studies that quantified SB proportion, a total of 6 [82,85,86,88,92,93] calculated proportion relative to wear or wake time, and 4 [78,79,81,89] calculated proportion relative to a full day (24 hours). Specifically, within CPM-based studies, using wear or wake time as a denominator yielded higher SB proportions (range: 57%-87.2%) compared to those calculated relative to a 24-hour day (range: 43.55%-72%).

### Algorithm Type

Of the 12 studies (16 cohorts) using proprietary algorithms, 8 reported SB proportion per day [78,79,81,85,86,89,92,93] (11 cohorts; range: 43.55%-89%), and 5 reported SB time [78,83,84,86,90] (range: 510-1203 minutes per day). Both studies using academic algorithms [76,77] reported SB time (range: 476.2-1224 minutes per day). The 2 studies using custom algorithms [80,88] also reported SB time (range: 565.6-622.2 minutes per day). No clear patterns emerged across algorithm types, as estimates overlapped considerably.

## Discussion

### Overview

This systematic review is the first to characterize SB across cognitive impairments (ie, MCI and dementia) and critically appraise the wearable digital methods used to measure it. A key finding is that while total SB volume did not consistently differ between those who are cognitively impaired and controls, their pattern appeared to. Specifically, individuals with dementia exhibited significantly fewer but longer sedentary bouts. However, this review also highlights significant methodological inconsistencies in how SB is measured. Variations in device type and placement, wear-time protocols, cut-point thresholds, proportion denominators, and reported outcomes limit cross-study comparisons and, consequently, our understanding of SB and its influencing factors.

### Impact of Cognitive Impairment and Disease Subtype on SB

To reflect Aim 1, this review suggests that the relationship between cognitive impairment and SB may be characterized less by a simple increase in volume and more by a potential shift in pattern. The available evidence indicates that people with dementia tend to exhibit longer and fewer sedentary bouts compared with those with MCI and cognitively intact controls [79,85,93], a profile that could be indicative of more prolonged, uninterrupted SB, which is often considered the most hazardous type [94]. However, no longitudinal research was identified in the review, meaning we cannot ascertain how SB patterns change with disease progression or if it predicts worse outcomes. Furthermore, our synthesis points to a nuanced conclusion: a significantly greater volume of SB does not appear to be a consistent hallmark of cognitive impairment. Among the 12 studies (comprising 18 cohorts) that considered control comparisons, only 4 identified a significant increase in SB volume [85,90,91,93]. Of these, 3 (75%) were specific to dementia cohorts, while 1 pertained to a group with mixed cognitive impairment. The existing self-report literature, which focuses on physical inactivity, provides a relevant context for interpreting the current findings on SB. Studies have found that physical inactivity is not directly associated with lower global cognition in community-dwelling adults with dementia [95-100]. Instead, inactivity appears to be more closely linked to other factors, including depression, a history of falls, fewer waking hours, and environmental barriers [95,99-102]. This suggests that cognitive impairment may not inevitably lead to pervasive inactivity, which may also extend to SB, pointing to the influence of other mediating factors.

The apparent discrepancy between inconsistent volume and consistent pattern findings may be explained by neuropsychiatric symptoms and their interaction with the environment, rather than the disease pathology itself. For instance, apathy, a prevalent symptom in dementia, varies significantly between individuals and may manifest not as a greater total sedentary time, but as a reduced frequency of interruption, leading to the observed pattern of prolonged bouts [32,103]. This perspective is supported by Alonzo’s [104] theory, which posits that the impact of an illness emerges from its interaction with the individual’s environment. In this view, symptoms like apathy and disorientation interact with environmental contexts (eg, institutional settings and lack of stimulation) to promote sedentariness. Qualitative evidence corroborates this mechanism, identifying symptoms such as exhaustion and disorientation as common barriers to activity [103]. Therefore, future qualitative research is warranted to further elucidate the lived experience of sedentariness in this population.

It is important to note, however, that this interpretation is constrained by the methodological limitations identified in this review. The widespread use of wrist-worn devices and unvalidated cut points [37,105] is likely to impair the detection of short, nonpostural movements that break up sedentary time. Therefore, the current evidence base may underestimate the true extent of sedentary patterns in cognitive impairment, and the potential role of symptoms warrants further investigation with more precise and standardized measurement tools.

Furthermore, the generalizability of these findings across dementia subtypes remains uncertain. The available evidence is predominantly derived from cohorts with Alzheimer disease (88% of studies that reported dementia subtypes). Other dementia subtypes were included but not analyzed separately [91,93], preventing insights into how their unique symptomatic profiles might influence distinct SB patterns [106,107]. The current literature’s focus on Alzheimer disease underscores a significant evidence gap regarding SB in non- Alzheimer disease dementias, which future research should devote attention to.

### Current Approaches for Wearable Digital Methods and Metrics for Characterizing SB in People With Cognitive Impairment

Aligned with Aim 2, key findings in this review indicate that, in keeping with existing literature [108], studies quantified SB through 2 distinct methods: identifying body posture or applying cut-point thresholds. These approaches also shape the type of outcomes that can be derived; posture-based methods more readily capture pattern-related metrics (eg, bouts and breaks), whereas cut-point threshold approaches primarily quantify overall sedentary volume. This methodological divergence is compounded by differences in device placement and detection mechanisms. For posture-based measurements, thigh-worn devices struggle to distinguish sitting from lying [43], while lower-back devices struggle to distinguish sitting from standing [109]. In contrast, cut-point thresholds do not distinguish between postures and are highly sensitive to nonambulatory arm movements. Consequently, wrist-worn devices may misclassify sedentary fidgeting as nonsedentary time [110,111].

This pattern is reflected in the findings of the present review: trunk placements (eg, hip, lower back, and waist) generally reported higher and more stable estimates of SB time and proportion, whereas wrist-worn devices produced the lowest SB proportions and the greatest variability in SB time. However, these differences may also be partially explained by how SB is characterized, as wrist-worn studies demonstrated greater variability in cut-point thresholds compared with more standardized placements such as the hip. Collectively, these findings suggest that differences in SB estimates may be driven less by device placement alone and more by underlying methodological choices (eg, CPM thresholds), which reflect differing conceptualizations of SB.

The CPM approach was the most common method adopted to measure SB in cognitive impairment, with 71% (12/17) of studies using this method. The CPM approach is widely used in SB research [112] and functions as a proxy measure for energy expenditure (METs) [40], aligning with the standard SB definition of ≤1.5 METs. However, its application is highly inconsistent; 6 different CPM thresholds were identified in this review, none of which were validated in cognitively impaired populations. Although 2 thresholds (178.5 CPM [78,81,90] and 1853 vector magnitude CPM [85]) were validated in healthy older adults, the remainder lacked validation in any older population, significantly undermining the validity and comparability of findings.

The consequences of this inconsistency were most apparent in estimates of sedentary proportion. Unlike the broader literature, where lower cut points typically yield lower sedentary estimates [43,113], studies in this review using lower thresholds (≤99–100 CPM) reported the highest sedentary proportions, whereas higher thresholds (≤178.5 CPM) produced lower estimates. This inverse pattern suggests that lower cut points validated in younger populations may overestimate sedentary time when applied to older adults with cognitive impairment, while thresholds derived from older populations may provide more accurate estimates. These trends were not observed for absolute sedentary time (minutes per day) or by algorithm type (proprietary, academic, and custom), indicating that proportional differences may instead reflect denominator choice. Studies using waking or wear-time denominators reported higher sedentary proportions than those using a 24-hour denominator, as excluding sleep and nonwear reduces the denominator and inflates the proportion of time classified as sedentary. This aligns with methodological evidence demonstrating that accelerometer processing decisions, including wear-time definitions, substantially influence sedentary estimates and complicate comparisons across studies [114-117]. Together, inconsistencies in cut-point selection and denominator choice limit comparability across studies and hinder evidence synthesis.

While less common, posture-based methods (used by 24% of studies) offer a more direct assessment of SB. This approach circumvents the need for population-specific energy expenditure validation and may offer more accurate detection of SB [24,43]. Matthews et al [29] have called for such objective definitions to incorporate posture, arguing that energy-based criteria alone may not fully capture the nature of SB. However, this method introduces a different set of limitations; crucially, it cannot estimate METs and is therefore limited to classifying posture rather than intensity [118]. This is a significant constraint, as it diverges from the consensus energy-based definition of SB (≤1.5 METs). Therefore, the definition provided by Chastin and Granat [24] (“any nonupright activity”) may offer a practical alternative. Thus, the choice of method to measure SB presents a fundamental trade-off: prioritizing conceptual alignment with the energy-based definition of SB, often via CPM, at the potential cost of measurement accuracy (due to the current lack of validated CPM thresholds), or opting for the practical accuracy of a posture-based definition (“any nonupright activity”) that may neglect the MET component of SB.

It is important to recognize that SB, like all behaviors, is multifaceted and challenging to fully capture with a single measurement [38]. Therefore, future research should carefully consider the limitations of existing measurement tools and select those best aligned with specific research objectives and target populations. Crucially, measures must adhere to core principles of good measurement: demonstrating reliability, validity, population specificity, and sensitivity to change [119,120]. By emphasizing such criteria, researchers can enhance the accuracy and relevance of SB assessment, particularly in populations with unique physiological profiles such as cognitive impairment.

### Recommendations

To aid future research and advance the field, we propose a set of recommendations to guide more meaningful and standardized approaches to measuring SB in people with cognitive impairment (Textbox 1).

**Recommendations for standardizing sedentary behavior measurement in cognitive impairment research.**

**Recommendations**

- Validate cut-point thresholds and/or body posture algorithms in older adults, or populations with cognitive impairment, where possible, ensuring they account for functional limitations and typical behavioral patterns for their application in people with cognitive impairment.
- Standardize the reporting of sedentary proportion. To support cross-study comparability, researchers should either:
  Report sedentary proportion using a fixed 24-hour denominator.
  If reporting proportion relative to a non–24-hour specified period (eg, wear or wake time), also report the absolute sedentary time (eg, total minutes per day) to allow readers and future analyses to calculate proportions using their preferred denominator.
- Establish minimum reporting standards for sedentary behavior methodology. Studies should explicitly report device model and placement, cut-point threshold, and validation source, denominator used for proportional estimates, wear-time criteria, and sleep inclusion/exclusion status.
- Develop and adopt a core set of outcomes for sedentary behavior research in cognitive impairment, including key metrics, such as total sedentary time, number of prolonged bouts, and mean bout length, to enable cross-study comparison.

### Strengths and Limitations

This review has several strengths. It is the first to systematically examine the wearable digital technology methods and metrics used to assess SB in individuals with cognitive impairment, offering a comprehensive overview of current practices and highlighting key methodological inconsistencies. The study also extends its scope to consider the influence of cognitive impairment severity and disease subtype on SB, which has received limited attention in previous research. A rigorous search strategy, transparent inclusion criteria, and quality appraisal of included studies further strengthen the reliability of the findings.

However, this review’s findings are subject to important limitations. The categorization of SB into volume, pattern, and variability, while necessary for synthesis and aligned with existing frameworks [72,121], may not capture all relevant dimensions of SB. Furthermore, the deliberate exclusion of home-based technologies, though methodologically justified by their current lack of standardized validation and restricted ecological scope [48,122], delimits the evidentiary basis exclusively to wearable-derived data. Consequently, the conclusions presented here should be interpreted within the context of these methodological boundaries.

### Conclusions

This review establishes that while preliminary evidence suggests that SB patterns differ across cognitive impairment, differences in SB volume are not consistently observed. Furthermore, the increasing use of wearable technology to assess SB in cognitive impairment is hampered by significant methodological heterogeneity and a critical lack of population-specific validation. This heterogeneity underscores the critical need for a consistent methodology to advance the field and establish robust, reproducible evidence on the role of SB in cognitive impairment.

## Data Availability

All data generated or analyzed during this study are included in this published article and its Multimedia Appendices 1-5. All articles included in the review are publicly available online. The complete search strategy, including the search strings used for each database, is provided in Multimedia Appendices 1-5 to facilitate reproducibility.

## Appendix

Multimedia Appendix 1 PRISMA checklist.

Multimedia Appendix 2 Search strategy: keywords and MeSH terms for systematic literature review.

Multimedia Appendix 3 Quality assessment of all studies included in this systematic review.

Multimedia Appendix 4 Comprehensive overview of study characteristics with main findings and comparisons.

Multimedia Appendix 5 Matrix of all studies and corresponding parameters used to describe sedentary behaviour.

## Abbreviations

AD: Alzheimer disease
CPM: counts per minute
MCI: mild cognitive impairment
MET: metabolic equivalent task
PA: physical activity
SB: sedentary behavior
