# Supplementary material for: Use of Wearable Technology for Measuring and Characterizing Sedentary Behavior in People With Mild Cognitive Impairment and Dementia: Systematic Review
Source: JMIR Aging. 2026 Jun 25;9:e85361. doi: 10.2196/85361 (PMC13351645; doi:10.2196/85361)
Supplement: Multimedia Appendix 2 [file aging_v9i1e85361_app2.docx]

| **Multimedia Appendix 2:** Search strategy: keywords and MeSH terms for systematic literature review. | | |
| --- | --- | --- |
| **Concept** | **Keywords^a^** | **MeSH Terms^b^** |
| 1. Cognitive Impairment | Dementia* OR “cognitive* impair*” OR “cognitive* dysfunction*” OR Alzheimer* OR Vascular* OR Lewy* OR MCI | **Medline:** dementia/, Alzheimer disease/, dementia, vascular/, dementia, multi-infarct/, lewy body disease/, mixed dementias/, cognitive dysfunction/.  **Embase:** cognitive defect/, dementia/, Alzheimer disease/, diffuse lewy body disease/, frontotemporal dementia/, mental deterioration/, mixed dementia/, multiinfarct dementia/, mild cognitive impairment/.  **Psycinfo:** mild cognitive impairment/, dementia/, alzheimer’s disease/, dementia with lewy bodies/, vascular dementia/, cognitive impairment/, frontotemporal dementia/. |
| 1. Sedentary Behaviour | Sedentar* OR inactive* OR “low energy expend*” OR “seat* posture” OR sitting OR reclin* OR “supine position” | **Medline:** sedentary behaviour/, sitting position/, supine position/.  **Embase:** sedentary lifestyle/, physical inactivity/, sitting/, supine position/.  **Psycinfo:** sedentary behavior/. |
| 1. Wearable Technologies | Wearable* OR smart* OR acceleromet* OR digital* OR gyroscop* OR inertial* OR magnetometer* OR tracker* OR inclinometer* OR motion* OR actimetry | **Medline:** Wearable electronic devices/, fitness trackers/, accelerometery/, actigraphy.  **Embase:** wearable technology/, accelerometry/, accelerometer/, actimetry/, inertial sensor/, magnetometer/, gyroscope sensor/, activity tracker/, smart watch/, wearable computer/, wrist-worn device/, wearable sensor/, wearable device/.  **Psycinfo:** wearable device/, actigraphy/. |
| *Note: ^a^:used in Medline, Embase, Psycinfo, Scopus and Web of Science, ^b^:used in Medline, Embase and Psycinfo, *: truncation symbol. Searches combined with AND: #1 AND #2 AND #3.* | | |
