# Supplementary material for: Use of Wearable Technology for Measuring and Characterizing Sedentary Behavior in People With Mild Cognitive Impairment and Dementia: Systematic Review
Source: JMIR Aging. 2026 Jun 25;9:e85361. doi: 10.2196/85361 (PMC13351645; doi:10.2196/85361)
Supplement: Multimedia Appendix 3 [file aging_v9i1e85361_app3.docx]

| 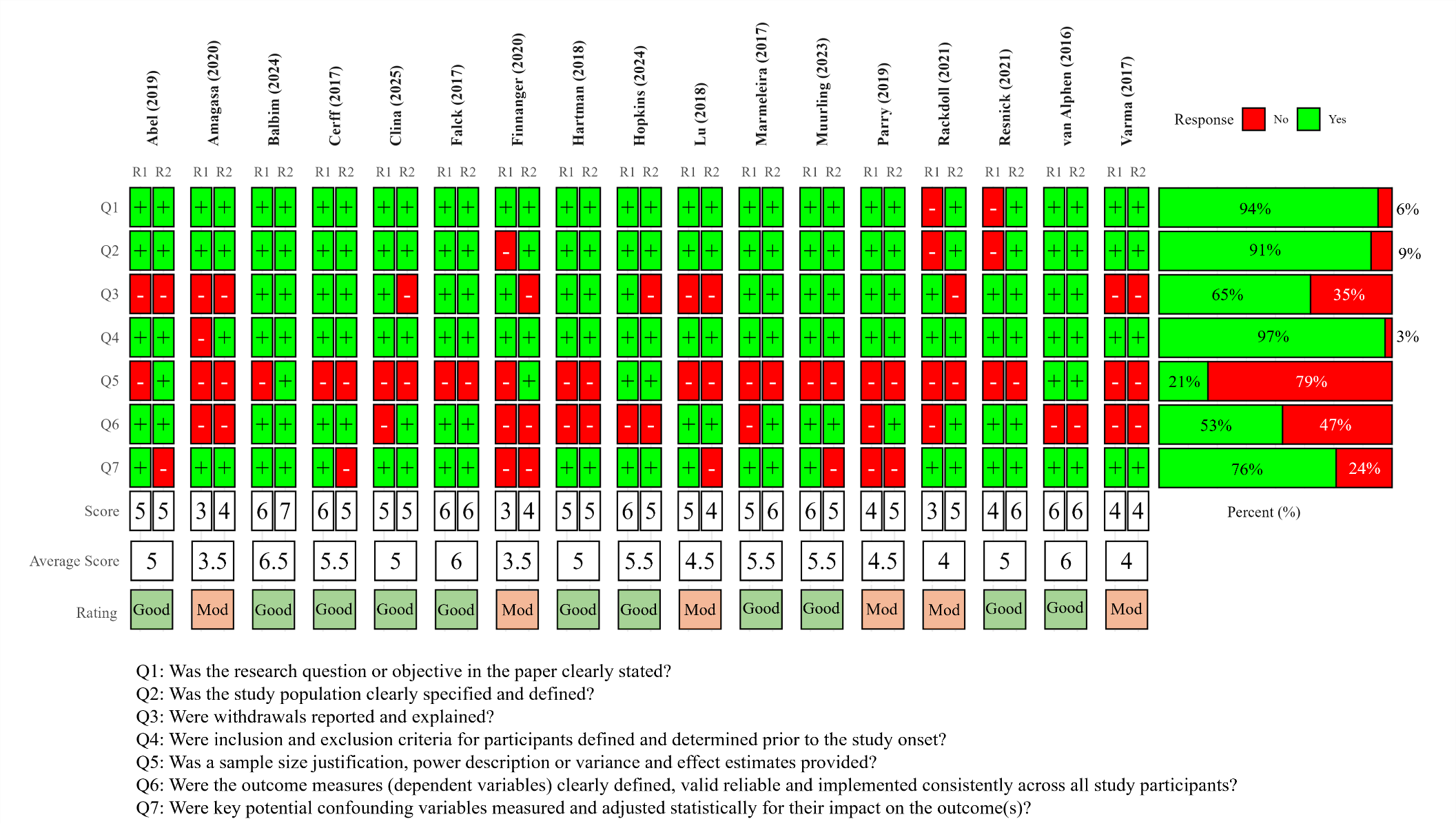 |
| --- |
| **Multimedia Appendix 3:** Quality assessment of all studies included in this systematic review.  *Note: Right bar graph shows proportion of responses per question. R1: Rater one; R2: Rater two; ‘+’: Yes; ‘-‘: No; Mod: Moderate* |

**References**

Abel, B., Pomiersky, R., Werner, C., Lacroix, A., Schäufele, M., & Hauer, K. (2019). Day-to-day variability of multiple sensor-based physical activity parameters in older persons with dementia. *Arch Gerontol Geriatr*, *85*, 103911. <https://doi.org/10.1016/j.archger.2019.103911>

Amagasa, S., Inoue, S., Murayama, H., Fujiwara, T., Kikuchi, H., Fukushima, N., Machida, M., Chastin, S., Owen, N., & Shobugawa, Y. (2020). Associations of Sedentary and Physically-Active Behaviors With Cognitive-Function Decline in Community-Dwelling Older Adults: Compositional Data Analysis From the NEIGE Study. *J Epidemiol*, *30*(11), 503-508. <https://doi.org/10.2188/jea.JE20190141>

Balbim, G. M., Falck, R. S., Boa Sorte Silva, N. C., Kramer, A. F., Voss, M., & Liu-Ambrose, T. (2024). The Association of the 24-Hour Activity Cycle Profiles With Cognition in Older Adults With Mild Cognitive Impairment: A Cross-Sectional Study. *J Gerontol A Biol Sci Med Sci*, *79*(7). <https://doi.org/10.1093/gerona/glae099>

Cerff, B., Maetzler, W., Sulzer, P., Kampmeyer, M., Prinzen, J., Hobert, M. A., Blum, D., van Lummel, R., Del Din, S., Gräber, S., Berg, D., & Liepelt-Scarfone, I. (2017). Home-Based Physical Behavior in Late Stage Parkinson Disease Dementia: Differences between Cognitive Subtypes. *Neurodegener Dis*, *17*(4-5), 135-144. <https://doi.org/10.1159/000460251>

Clina, J. G., Bodde, A. E., Chang, J., Helsel, B. C., Sherman, J. R., Vidoni, E. D., Williams, K. N., Washburn, R. A., Donnelly, J. E., & Ptomey, L. T. (2025). Factors Associated With Physical Activity in Alzheimer’s Disease: A Cross-Sectional Study of Individuals and Their Caregivers. *Journal of Aging and Health*, *0*(0), 08982643251318766. <https://doi.org/10.1177/08982643251318766>

Falck, R. S., Landry, G. J., Best, J. R., Davis, J. C., Chiu, B. K., & Liu-Ambrose, T. (2017). Cross-Sectional Relationships of Physical Activity and Sedentary Behavior With Cognitive Function in Older Adults With Probable Mild Cognitive Impairment. *Physical Therapy*, *97*(10), 975-984. <https://doi.org/10.1093/ptj/pzx074>

Finnanger Garshol, B., Ellingsen-Dalskau, L. H., & Pedersen, I. (2020). Physical activity in people with dementia attending farm-based dementia day care – a comparative actigraphy study. *BMC Geriatrics*, *20*(1), 219. <https://doi.org/10.1186/s12877-020-01618-4>

Hartman, Y. A. W., Karssemeijer, E. G. A., van Diepen, L. A. M., Olde Rikkert, M. G. M., & Thijssen, D. H. J. (2018). Dementia Patients Are More Sedentary and Less Physically Active than Age- and Sex-Matched Cognitively Healthy Older Adults. *Dement Geriatr Cogn Disord*, *46*(1-2), 81-89. <https://doi.org/10.1159/000491995>

Hopkins, J., McVeigh, J., Hill, K., Ellis, K. A., Jacques, A., & Burton, E. (2024). Associations between physical activity, sedentary behaviour and cognitive domain performance of people living with mild cognitive impairment in the community. *Aust Occup Ther J*, *71*(4), 527-539. <https://doi.org/10.1111/1440-1630.12944>

Lu, Z., Harris, T. B., Shiroma, E. J., Leung, J., & Kwok, T. (2018). Patterns of Physical Activity and Sedentary Behavior for Older Adults with Alzheimer's Disease, Mild Cognitive Impairment, and Cognitively Normal in Hong Kong. *J Alzheimers Dis*, *66*(4), 1453-1462. <https://doi.org/10.3233/jad-180805>

Marmeleira, J., Ferreira, S., & Raimundo, A. (2017). Physical activity and physical fitness of nursing home residents with cognitive impairment: A pilot study. *Exp Gerontol*, *100*, 63-69. <https://doi.org/10.1016/j.exger.2017.10.025>

Muurling, M., Badissi, M., de Boer, C., Legdeur, N., Barkhof, F., van Berckel, B. N. M., Maier, A. B., Pijnappels, M., & Visser, P. J. (2023). Physical activity levels in cognitively normal and cognitively impaired oldest-old and the association with dementia risk factors: a pilot study. *BMC Geriatrics*, *23*(1), 129. <https://doi.org/10.1186/s12877-023-03814-4>

Parry, S., Chow, M., Batchelor, F., & Fary, R. E. (2019). Physical activity and sedentary behaviour in a residential aged care facility. *Australas J Ageing*, *38*(1), E12-e18. <https://doi.org/10.1111/ajag.12589>

Rackoll, T., Neumann, K., Passmann, S., Grittner, U., Külzow, N., Ladenbauer, J., & Flöel, A. (2021). Applying time series analyses on continuous accelerometry data-A clinical example in older adults with and without cognitive impairment. *PLoS One*, *16*(5), e0251544. <https://doi.org/10.1371/journal.pone.0251544>

Resnick, B., Boltz, M., Galik, E., Fix, S., & Zhu, S. (2021). Feasibility, Reliability, and Validity of the MotionWatch 8 to Evaluate Physical Activity Among Older Adults With and Without Cognitive Impairment in Assisted Living Settings. *J Aging Phys Act*, *29*(3), 391-399. <https://doi.org/10.1123/japa.2020-0198>

van Alphen, H. J., Volkers, K. M., Blankevoort, C. G., Scherder, E. J., Hortobágyi, T., & van Heuvelen, M. J. (2016). Older Adults with Dementia Are Sedentary for Most of the Day. *PLoS One*, *11*(3), e0152457. <https://doi.org/10.1371/journal.pone.0152457>

Varma, V. R., & Watts, A. (2017). Daily Physical Activity Patterns During the Early Stage of Alzheimer's Disease. *J Alzheimers Dis*, *55*(2), 659-667. <https://doi.org/10.3233/jad-160582>
