# Supplementary material for: Use of Wearable Technology for Measuring and Characterizing Sedentary Behavior in People With Mild Cognitive Impairment and Dementia: Systematic Review
Source: JMIR Aging. 2026 Jun 25;9:e85361. doi: 10.2196/85361 (PMC13351645; doi:10.2196/85361)
Supplement: Multimedia Appendix 4 [file aging_v9i1e85361_app4.docx]

| **Multimedia Appendix 4:** Comprehensive Overview of Study Characteristics with Main Findings and Comparisons. | | | | |
| --- | --- | --- | --- | --- |
| **1^st^ Author (Year), Country, Study Design, Study Quality** | **Participant Demographics** | **Data Collection, Processing and Analysis** | **Main Study Findings** | **Comparisons** |
| Abel (2019) [1]  **Country:** Germany  **Study Design:** Baseline recruits from RCT  **Study Quality:** Good | **53 Dementia:**  Disease Subtype: Not specified  Cognition Severity: Mild-Moderate  Age: 82.3±5.6 years  Sex (M/F): 14/39  Dwelling status (CD/IT): 15/38  MMSE: 22.1±2.9 | **Collection:** Accelerometer (PAMSys, PAMSys, Biosenics) worn on chest for 3 days (Friday-Sunday)  **Non-Wear Identification:** Not specified.  **Valid Wear Criteria:** Not specified.  **SB Classification:** Body posture  **SB Algorithm:** Academic. | **Lying time:** 9.1 hrs/day  **Sitting time:** 11.3 hrs/day  **Inactive time:** 20.4 hrs/day  **Day-to-day variability:** Low day-to-day variability between Friday-Saturday (ICC: 0.82-0.85) and Saturday-Sunday (ICC: 0.83-0.85). | No Control Comparisons |
| Amagasa (2020) [2]  **Country:** Japan  **Study Design:** Cross-sectional  **Study Quality:** Moderate | **48 Mixed Cognitive Impairment:**  Disease Subtypes: MCI & AD  Cognition Severity: Not specified  Age: 77.6±5.4 years  Sex (M/F): 23/25  Dwelling status (CD/IT): 48/0  **463 Controls:**  Age: 73±5.4 years  Sex: 217/246  Dwelling status (CD/IT): 463/0 | **Collection:** Accelerometer (Active style Pro HJA-750C, Omron) worn on waist for 7 consecutive days, whilst awake (except water-based activities).  **Non-Wear Identification:** ≥60 min zero counts.  **Valid Wear Criteria:** ≥4 days, ≥10 hours/day  **SB Classification:** ≤1.5 METs  **SB Algorithm:** Academic. | **SB time:**   - CI: 476.2 min/day - Controls: 442.4 min/day | - SB time did not significantly differ between CI and controls (P=0.086). |
| Balbim (2024) [3]  **Country:** Canada  **Study Design:** Cross-Sectional  **Study Quality:** Good | **253 MCI:**  Disease Subtype: Not specified  Cognition Severity: Not specified  Age: 73.69±5.41 years  Sex (M/F): 96/157  Dwelling status (CD/IT): 253/0  MMSE: 27.73±1.9  MoCA: 22.05±2.85 | **Collection:** Actigraph (MotionWatch8, CamNtech) worn on non-dominant wrist for 7 consecutive days.    **Non-Wear Identification:** Did not perform.  **Valid Wear Criteria:** ≥5 days (hours not reported).  **SB Classification:** ≤178.5 CPM  **SB Algorithm:** Proprietary (MotionWare). | **SB time:** 626.9 min/day  **SB proportion (% 24 hrs):** 43.55%/day | No Control Comparisons |
| **1^st^ Author (Year), Country, Study Design, Study Quality** | **Participant Demographics** | **Data Collection, Processing and Analysis** | **Main Study Findings** | **Comparisons** |
| Cerff (2017) [4]  **Country:** Germany  **Study Design:** Cross-Sectional  **Study Quality:** Good | **22 MCI:**  Disease Subtype: PDD-MCI  Cognition Severity: Not specified  Age: 68 (57-78) years  Sex (M/F): 17/5  Dwelling status (CD/IT): 22/0  UPDRS-III: 24 (10-62)  **9 Dementia:**  Disease Subtype: PDD  Cognition Severity: Mild-Severe  Age: 72 (67-75) years  Sex (M/F): 9/0  Dwelling status (CD/IT): 9/0  UPDRS-III: 36 (14-56)  **17 Controls:**  Age: 71 (44-88) years  Sex (M/F): 10/7  Dwelling status (CD/IT): 17/0  UPDRS-III: 20 (11-58) | **Collection:** Accelerometer (Dynaport MiniMod, McRoberts) worn on lower-back for 3 consecutive days.  **Non-Wear Identification:** Proprietary (McRoberts).  **Valid Wear Criteria:** 24 hours/day or >80% of 24 hours (no day minimum specified).  **SB Classification:** Body posture.  **SB Algorithm:** Proprietary (McRoberts). | **SB proportion (% 24 hrs):**   - PDD-MCI: 78%/day - PDD: 89%/day - Controls: 75%/day   **Lying proportion (% 24 hrs):**   - PDD-MCI: 42%/day - PDD: 40%/day - Controls: 37%/day   **Sitting proportion (% 24 hrs):**   - PDD-MCI: 34%/day - PDD: 42%/day - Controls: 36%/day   **SB proportion ≤ 1.5 METs (% 24 hrs):**   - PDD-MCI: 84%/day - PDD: 89%/day - Controls: 83%/day   **Number of SB bouts:**   - PDD-MCI: 129 bouts/day - PDD: 97 bouts/day - Controls: 134 bouts/day   **Mean SB bout length:**   - PDD-MCI: 515 sec - PDD: 727 sec - Controls: 506 sec | - PDD engaged in significantly fewer SB bouts vs PDD-MCI and controls (P=0.01). - Mean sedentary bout length was significantly longer in PDD vs PDD-MCI and controls (P=0.02). |
| Clina (2025) [5]  **Country:** USA  **Study Design:** Cross-Sectional  **Study Quality:** Good | **65 Dementia:**  Disease Subtype: ADRD  Cognition Severity: Mild-Moderate  Age: 73.6±8.4 years  Sex (M/F): 41/24  Dwelling status (CD/IT): 65/0  Ethnicity: 96.9% White  **65 Controls (Caregivers):**  Age: 69.4 ± 9.4 years  Sex (M/F): 20/45  Dwelling status (CD/IT): 65/0  Ethnicity: 96.9% White | **Collection:** Accelerometer (wGT3x-BT, ActiGraph) worn during waking hours on non-dominant hip for 7 days (except water-based activities).  **Non-Wear Identification:**  ≥90 min zero counts  **Valid Wear Criteria:** ≥3 days, ≥10 hours/day (including 1 weekend day).  **SB Classification:** ≤100 CPM  **SB Algorithm:** Custom. | **SB time:**   - Dementia (ADRD): 622.2 mins/day - Controls: 591.8 mins/day | - No significant differences found between SB time of dementia (ADRD) participants and controls. |
| **1^st^ Author (Year), Country, Study Design, Study Quality** | **Participant Demographics** | **Data Collection, Processing and Analysis** | **Main Study Findings** | **Comparisons** |
| Falck (2017) [6]  **Country:** Canada  **Study Design:** Cross-Sectional  **Study Quality:** Good | **81 MCI**  Disease Subtype: Not specified  Cognition Severity: Probable MCI  Age: 72.54±7.62 years  Sex (M/F): 33/48  Dwelling status (CD/IT): 81/0  MMSE: 28.65±1.15  MoCA: 11.84±2.11  **69 Controls**  Age: 69.42 ±6.37 years  Sex (M/F): 15/54  Dwelling status (CD/IT): 69/0  MMSE: 29.22±.87  MoCA: 27.19±1.1 | **Collection:** Actigraph (MotionWatch8, CamNtech) worn on non-dominant wrist for ≥4 days.  **Non-Wear Identification:**  ≥120 min zero counts.  **Valid Wear Criteria:** ≥4 days (hours not reported).  **SB Classification:** ≤178.5 CPM  **SB Algorithm:** Proprietary (MotionWare). | **SB proportion (% 24 hrs):**   - MCI: 61.65 %/day - Controls: 57.24/day   **SB bouts (≥30 min):**   - MCI: 4.07 bouts/day - Controls: 3.30 bouts/day | - SB proportion (%) did not significantly differ between MCI and controls (P=0.161). - MCI participants engaged in significantly more 30+ minute SB bouts per day vs controls (P=0.046). |
| Finnanger (2020) [7]  **Country:** Norway  **Study Design:** Cross-Sectional  **Study Quality:** Moderate | **29 Dementia (FDC):**  Disease Subtype: Not specified  Cognition Severity: Mild-Severe  Age: 74±7.22 years  Sex (M/F): 20/9  Dwelling status (CD/IT): 29/0  CDR: 1.22±0.57  **107 Dementia (RDC):**  Disease Subtype: Not specified  Cognition Severity: Mild-Severe  Age: 84.3±8.1 years  Sex (M/F): 36/71  Dwelling status (CD/IT): 107/0  CDR: 1.53±0.67 | **Collection:** Actigraph (Actisleep+, ActiGraph) worn on left wrist continuously for 7 consecutive days.  **Non-Wear Identification:**  Academic algorithm (Troiano 2007)  **Valid Wear Criteria:** ≥3 days, ≥8 hours/day (filtered 08:00-20:00)  **SB Classification:** ≤99 CPM  **SB Algorithm:** Proprietary (ActiLife). | **SB proportion (% wear time):**   - FDC: 39.7%/week - RDC: 43.51%/week | - No significant differences found in weekly SB proportion between farm-based dementia care participants (FDC) and regular day care participants (RDC) (P=0.209). |
| **1^st^ Author (Year), Country, Study Design, Study Quality** | **Participant Demographics** | **Data Collection, Processing and Analysis** | **Main Study Findings** | **Comparisons** |
| Hartman (2018) [8]  **Country:** The Netherlands  **Study Design:** Cross-Sectional  **Study Quality:** Good | **45 Dementia:**  Disease Subtypes: AD (n=25, 55.6%); VaD (n=2, 4.4%); ADV (n=12, 26.7%); Not Specified (n=6, 13.3%).  Cognition Severity: Unspecified  Age: 79.6±5.9 years  Sex (M/F): 23/22  Dwelling status (CD/IT): 42/3  MMSE: 22.8±3.2  **49 Controls:**  Age: 80±7.7 years  Sex (M/F): 24/25  Dwelling status (CD/IT): 48/1  MMSE: 29±1.2 | **Collection:** Actigraph (Actiwatch 2, Philips) worn on non-dominant wrist 24hours a day for 7 days.  **Non-Wear Identification:** Did not perform.  **Valid Wear Criteria:** ≥6 days, ≥10 hours/day.  **SB Classification:** <145 CPM  **SB Algorithm:** Proprietary (Philips Actiware 6). | **SB time:**   - Dementia: 8.5 hours/day - Controls: 8.2 hours/day   **SB proportion (% wake time):**   - Dementia: 57%/day - Controls: 55%/day   **SB interruptions:**   - Dementia: 27.2 interruptions/day - Controls: 28.2 interruptions/day   **SB bouts (≥30 min):**   - Dementia: 2.3 bouts/day - Controls: 2.0 bouts/day   **Mean SB bout length:**   - Dementia: 18.3 min - Controls: 16.6 min | - SB time did not significantly differ between dementia participants and controls (P=0.216). - Proportion of SB was significantly higher in dementia participants vs controls (P=0.042). - Number of SB interruptions did not significantly differ between dementia and controls (P=0.195). - Number of 30+ min SB bouts did not significantly differ between dementia and controls (P=0.227). - Duration of SB bouts was significantly longer in dementia participants vs controls (P=0.008). |
| Hopkins (2024) [9]  **Country:** Australia  **Study Design:** Cross-Sectional  **Study Quality:** Good | **82 MCI:**  Disease Subtype: Not specified  Cognition Severity: Not specified  Age: 72 (54-76) years  Sex (M/F): 36/46  Dwelling status (CD/IT): 82/0  MoCA: 24 (22-26) | **Collection:** Accelerometer (ActivPAL, PAL Technologies) worn on right thigh for 7 days.  **Non-Wear Identification:** Not specified.  **Valid Wear Criteria:** ≥10 hours/day (no day minimum specified).  **SB Classification:** Body posture.  **SB Algorithm:** Proprietary (PALanalysis). | **SB time:** 637 min/day  **Sitting time:** 558 min/day  **Lying time:** 21 min/day  **SB bouts (≥30 min):** 5 bouts/day  **SB bouts (≥60 min):** 1 bout/day | No Control Comparisons |
| **1^st^ Author (Year), Country, Study Design, Study Quality** | **Participant Demographics** | **Data Collection, Processing and Analysis** | **Main Study Findings** | **Comparisons** |
| Lu (2018) [10]  **Country:** Hong Kong  **Study Design:** Cross-Sectional  **Study Quality:** Moderate | **105 MCI:**  Disease Subtype: Not specified  Cognition Severity: Not specified  Age: 83.6±3.7 years  Sex (M/F): 54/51  Dwelling status (CD/IT): 105/0  MoCA: 18.1±3  **252 Cognitive Impairment:**  Disease Subtype: Not specified  Cognition Severity: Low MoCA Score  Age: 83.4±4 years  Sex (M/F): 132/120  Dwelling status (CD/IT): 248/4  MoCA: 19.1±3.1  **182 Dementia:**  Disease Subtype: AD  Cognition Severity: Not specified  Age: 80.8±5.9 years  Sex (M/F): 61/121  Dwelling status (CD/IT): 179/3  MoCA: 13.1±5.1  **271 Controls:**  Age: 81.9±3.5 years  Sex (M/F): 167/104  Dwelling status (CD/IT): 271/0  MoCA: 24.4±2.4 | **Collection:** Accelerometer (wGT3x-BT, ActiGraph) worn on non-dominant wrist all day for 7 days (except water-based activities).  **Non-Wear Identification:** ≥90 min zero counts.  **Valid Wear Criteria:** ≥3 days, ≥10 hours/day.  **SB Classification:** <1,853 VM CPM  **SB Algorithm:** Proprietary (ActiLife). | **SB Proportion (% wear time):**   - MCI: 57.1%/day - Low MoCA: 57.8%/day - Dementia (AD): 63.2%/day - Controls: 58.4%/day   **Mean SB bout length:**   - MCI: 6.3 min - Low MoCA: 6.5 min - Dementia (AD): 7.9 min - Controls: 6.6 min   **Number of SB bouts:**   - MCI: 89.4 bouts/day - Low MoCA: 91.4 bouts/day - Dementia (AD): 86.1 bouts/day - Controls: 91.5 bouts/day   **SB bouts (≥30 min):**   - MCI: 3.5 bouts/day - Low MoCA: 3.3 bouts/day - Dementia (AD): 4.1 bouts/day - Controls: 3.3 bouts/day | - Proportion of SB was significantly higher in dementia (AD) participants vs MCI, Low MoCA and controls (P<0.05). - Average SB bout length was significantly longer in dementia (AD) participants vs MCI, Low MoCA and controls (P<0.05). - Dementia participants (AD) engaged in significantly fewer SB bouts vs Low MoCA and controls (P<0.05). - Dementia participants engaged in significantly more SB bouts (≥30min) vs MCI, Low MoCA and controls (P<0.05). |
| Marmeleira (2017) [11]  **Country:** Portugal  **Study Design:** Cross-Sectional  **Study Quality:** Good | **48 Cognitive Impairment:**  Disease Subtype: Unspecified  Cognition Severity: Not specified  Age: 83.9 ±7.7 years  Sex (M/F): 13/35  Dwelling status (CD/IT): 0/48  MMSE: 14.9±4.9  **22 Controls:**  Age: 82.2 ±8.8 years  Sex (M/F): 10/12  Dwelling status (CD/IT): 0/22  MMSE: 25.8±2.2 | **Collection:** Accelerometer (GT1M, ActiGraph) worn on the right hip during daytime hours for 7 consecutive days.  **Non-Wear Identification:** ≥60 min zero counts.  **Valid Wear Criteria:** ≥3 days, ≥8 hours/day (filtered 07:00-20:00)  **SB Classification:** ≤100 CPM  **SB Algorithm:** Proprietary (ActiLife). | **SB time:**   - CI: 603.7 min/day - Controls: 601.0 min/day   **SB Proportion (% wear time):**   - CI: 87.2%/day - Controls: 84.0%/day | - No significant differences found in the volume of sedentary time or proportion between CI and controls. - CI were significantly more sedentary (P< 0.05) than controls during the hours of 7am-11am, 1pm and 4pm. |
| **1^st^ Author (Year), Country, Study Design, Study Quality** | **Participant Demographics** | **Data Collection, Processing and Analysis** | **Main Study Findings** | **Comparisons** |
| Muurling (2023) [12]  **Country:** The Netherlands  **Study Design:** Cross-Sectional  **Study Quality:** Good | **12 Mixed Cognitive Impairment**  Disease Subtype: MCI (n=4); AD (n=8)  Cognition Severity: Not specified.  Age: 91.7 ±2.6 years  Sex (M/F): 4/8  Dwelling status (CD/IT): 10/2  MMSE: 23.9±3.1  **49 Controls:**  Age: 92.5 ±1.8 years  Sex (M/F): 27/22  Dwelling status (CD/IT): 46/3  MMSE: 28.6±1.3 | **Collection:** Accelerometer (MoveMonitor, McRoberts) worn on the lower-back all day for 7 days (except water-based activities).  **Non-Wear Identification:** Proprietary (McRoberts).  **Valid Wear Criteria:** ≥75% of day average over 7 days; ≥4 days with ≥94% wear.  **SB Classification:** Body posture.  **SB Algorithm:** Proprietary (McRoberts). | **SB time:**   - CI: 142.7 hours/week - Controls: 140.7 hours/week   **Number of sitting bouts:**   - CI: 681.5 bouts/week - Controls: 741.1 bouts/week | - No significant differences found in the volume of SB time (hours/week) between CI and controls (P=0.40) - No significant differences found in the number of sitting bouts between CI and controls (P=0.30). |
| Parry (2019) [13]  **Country:** Australia  **Study Design:** Cross-Sectional  **Study Quality:** Moderate | **28 participants (sub-group demographic stratification unavailable):**  Age: 83.1 ±8.6 years  Sex (M/F): 20/8  Dwelling status (CD/IT): 0/28  **8 Dementia:**  Disease Subtype: Unspecified  Cognition Severity: Not specified  **20 Controls** | **Collection:** Accelerometer (GT3X, ActiGraph) worn during daytime hours (except water-based activities) for 5 consecutive days. Attached to the right hip of dementia participants and right thigh of controls.  **Non-Wear Identification:** ≥180 min zero counts.  **Valid Wear Criteria:** ≥5 days, ≥500 mins/day.  **SB Classification:** ≤100 CPM  **SB Algorithm:** Custom. | **SB time:**   - Dementia: 565.6 min/day - Controls: 583.9 min/day   **SB Proportion (% wear time):**   - Dementia: 86%/day - Controls: 85%/day | No Control Comparisons |
| **1^st^ Author (Year), Country, Study Design, Study Quality** | **Participant Demographics** | **Data Collection, Processing and Analysis** | **Main Study Findings** | **Comparisons** |
| Rackdoll (2021) [14]  **Country:** Germany  **Study Design:** Baseline data from intervention study  **Study Quality:** Moderate | **18 MCI:**  Disease Subtype: Not specified  Cognition Severity: Not specified  Age: 70 ±8 years  Sex (M/F): 8/10  Dwelling status (CD/IT): 18/0  MMSE: 28±1  **48 Controls:**  Age: 65 ±6 years  Sex (M/F): 23/25  Dwelling status (CD/IT): 48/0  MMSE: 29±1 | **Collection:** Accelerometer (GT3X+, ActiGraph) worn on hip at all times for 7 consecutive days (except water-based activities).  **Non-Wear Identification:** Not specified.  **Valid Wear Criteria:** Not specified.  **SB Classification:** ≤99 CPM  **SB Algorithm:** Proprietary (ActiLife). | **SB Proportion (% 24 hrs):**   - MCI: 72%/day - Controls: 74%/day | - No significant differences found in the SB time of MCI participants vs controls (SMD = 0.32). |
| Resnick (2021) [15]  **Country:** USA  **Study Design:** Baseline data from intervention study  **Study Quality:** Good | **781 participants (sub-group demographic stratification unavailable):**  Age: 89.48±7.43 years  Sex (M/F): 233/561  Dwelling status (CD/IT): 0/781  Ethnicity: 97% White  Mini-Cog: 2.39±0.76  **279 Cognitive Impairment:**  Disease Subtype: Unspecified  Cognition Severity: Not specified  **101 Controls** | **Collection:** Actigraph (MotionWatch8, CamNtech) worn on the wrist for 5 days.  **Non-Wear Identification:**  <1000 counts/day.  **Valid Wear Criteria:** Not specified.  **SB Classification:** ≤178.5 CPM  **SB Algorithm:** Proprietary (MotionWare). | **SB time (Day 2 outcomes):**   - CI: 1203 min/day - Controls: 1138 min/day | - CI participants spent significantly more time in SB on day 2 of assessment compared to controls (P=0.005). |
| **1^st^ Author (Year), Country, Study Design, Study Quality** | **Participant Demographics** | **Data Collection, Processing and Analysis** | **Main Study Findings** | **Comparisons** |
| van Alphen (2016) [16]  **Country:** The Netherlands  **Study Design:** Cross-Sectional  **Study Quality:** Good | **37 Dementia (Community-Dwelling):**  Disease Subtypes: AD (48.6%); VaD (16.2%); ADV (10.8%); DLB (8.1%); FTD (2.7%); PDD (5.4%); KD (2.7%); Unspecified (5.4%).  Cognition Severity: Not specified  Age: 77.3±5.6 years  Sex (M/F): 22/15  Dwelling status (CD/IT): 37/0  MMSE: 20.8±4.8  **83 Dementia (Institutionalised):**  Disease Subtypes: AD (49.4%); VaD (14.5%); ADV (9.6%); FTD (6%); KD (2.4%); Unspecified (18.1%)  Cognition Severity: Not specified  Age: 83±7.6 years  Sex (M/F): 17/66  Dwelling status (CD/IT): 0/83  MMSE: 15.5±6.5  **26 Controls:**  Age: 79.5±5.6 years  Sex (M/F): 13/13  Dwelling status (CD/IT): 26/0  MMSE: 28.2±1.6 | **Collection:** Actigraph (Actiwatch, AW-4, CamNtech) worn on the non-dominant wrist for >6 days.  **Non-Wear Identification:** Not specified.  **Valid Wear Criteria:** ≥6 days, 24 hours/day.  **SB Classification:** ≤100 CPM  **SB Algorithm:** Not specified. | **SB time:**   - IT Dementia (all, n=83): 17.3 hrs/day - IT Dementia (AD/ADV, n= 49): 16.82 hrs/day - IT Dementia (other dementia types, n=34): 18.0 hrs/day - CD Dementia (all, n=37): 15.83 hrs/day - CD Dementia (AD/ADV, n=22): 15.83 hrs/day - CD Dementia (other dementia types, n=15): 16.60 hrs/day - Controls: 14.52 hrs/day. | - SB time was significantly higher in dementia participants compared to controls (P<0.001). - IT dementia participants spent significantly more time (9.3% more) in SB compared to CD dementia (P=0.032) and controls (19% more, P<0.001). - AD participants spent significantly less time (6.9% less) in SB vs non-AD participants (P=0.047). |
| Varma (2017) [17]  **Country:** USA  **Study Design:** Cross-Sectional  **Study Quality:** Moderate | **39 Dementia:**  Disease Subtype: AD  Cognition Severity: Mild  Age: 73.5±7.9 years  Sex (M/F): 28/11  Dwelling status (CD/IT): 39/0  Ethnicity: 87.2% White  **53 Controls:**  Age: 73.2±6.5 years  Sex (M/F): 16/37  Dwelling status (CD/IT): 53/0  Ethnicity: 96.2% White | **Collection:** Accelerometer (GT3X+, ActiGraph) worn on the hip 24 hours a day for 7 days.  **Non-Wear Identification:** ≥90 min zero counts (Choi algorithm 2011, 2012).  **Valid Wear Criteria:** ≥10 hours/day (no day minimum specified).  **SB Classification:** ≤149 CPM  **SB Algorithm:** Proprietary (ActiLife). | **SB Proportion (% wake time):**   - Dementia (AD): 60.94%/day - Controls: 54.07%/day | - No significant differences found in SB volume between dementia and controls (P=0.830). |
| **Note:** Results displayed as mean ± standard deviation, or median (range) or displayed as ratio. **Abbreviations:** AD: Alzheimer’s Disease; ADRD: Alzheimer’s Disease Related Dementia; ADV: Alzheimer’s Disease with Vascular problems; CD: Community-Dwelling; CDR: Clinical Dementia Rating Scale; CI: Cognitive Impairment; CPM: Counts Per Minute; DLB: Dementia with Lewy Bodies; FDC: Farm-based Dementia Care; FTD: Frontotemporal Dementia; ICC: Intraclass Correlation; IT: Institutionalised dwelling; KD: Korsakoff Dementia; M/F: Male/Female Ratio; MCI: Mild Cognitive Impairment; METs: Metabolic Equivalent Tasks; MMSE: Mini Mental State Examination; MoCA: Montreal Cognitive Assessment; PDD: Parkinson’s Disease Dementia; PDD-MCI: Parkinson’s Disease Mild Cognitive Impairment; RCT: Randomised Control Trial; RDC: Regular Day Care; SB: Sedentary Behaviour; UPDRS-III: Unified Parkinson Disease Rating Scale Part III; USA: United States of America; VaD: Vascular Dementia; VM: Vector Magnitude. | | | | |

References

1. Abel, B., et al., *Day-to-day variability of multiple sensor-based physical activity parameters in older persons with dementia.* Arch Gerontol Geriatr, 2019. **85**: p. 103911.

2. Amagasa, S., et al., *Associations of Sedentary and Physically-Active Behaviors With Cognitive-Function Decline in Community-Dwelling Older Adults: Compositional Data Analysis From the NEIGE Study.* J Epidemiol, 2020. **30**(11): p. 503-508.

3. Balbim, G.M., et al., *The Association of the 24-Hour Activity Cycle Profiles With Cognition in Older Adults With Mild Cognitive Impairment: A Cross-Sectional Study.* J Gerontol A Biol Sci Med Sci, 2024. **79**(7).

4. Cerff, B., et al., *Home-Based Physical Behavior in Late Stage Parkinson Disease Dementia: Differences between Cognitive Subtypes.* Neurodegener Dis, 2017. **17**(4-5): p. 135-144.

5. Clina, J.G., et al., *Factors Associated With Physical Activity in Alzheimer’s Disease: A Cross-Sectional Study of Individuals and Their Caregivers.* Journal of Aging and Health, 2025. **0**(0): p. 08982643251318766.

6. Falck, R.S., et al., *Cross-Sectional Relationships of Physical Activity and Sedentary Behavior With Cognitive Function in Older Adults With Probable Mild Cognitive Impairment.* Physical Therapy, 2017. **97**(10): p. 975-984.

7. Finnanger Garshol, B., L.H. Ellingsen-Dalskau, and I. Pedersen, *Physical activity in people with dementia attending farm-based dementia day care – a comparative actigraphy study.* BMC Geriatrics, 2020. **20**(1): p. 219.

8. Hartman, Y.A.W., et al., *Dementia Patients Are More Sedentary and Less Physically Active than Age- and Sex-Matched Cognitively Healthy Older Adults.* Dement Geriatr Cogn Disord, 2018. **46**(1-2): p. 81-89.

9. Hopkins, J., et al., *Associations between physical activity, sedentary behaviour and cognitive domain performance of people living with mild cognitive impairment in the community.* Aust Occup Ther J, 2024. **71**(4): p. 527-539.

10. Lu, Z., et al., *Patterns of Physical Activity and Sedentary Behavior for Older Adults with Alzheimer's Disease, Mild Cognitive Impairment, and Cognitively Normal in Hong Kong.* J Alzheimers Dis, 2018. **66**(4): p. 1453-1462.

11. Marmeleira, J., S. Ferreira, and A. Raimundo, *Physical activity and physical fitness of nursing home residents with cognitive impairment: A pilot study.* Exp Gerontol, 2017. **100**: p. 63-69.

12. Muurling, M., et al., *Physical activity levels in cognitively normal and cognitively impaired oldest-old and the association with dementia risk factors: a pilot study.* BMC Geriatrics, 2023. **23**(1): p. 129.

13. Parry, S., et al., *Physical activity and sedentary behaviour in a residential aged care facility.* Australas J Ageing, 2019. **38**(1): p. E12-e18.

14. Rackoll, T., et al., *Applying time series analyses on continuous accelerometry data-A clinical example in older adults with and without cognitive impairment.* PLoS One, 2021. **16**(5): p. e0251544.

15. Resnick, B., et al., *Feasibility, Reliability, and Validity of the MotionWatch 8 to Evaluate Physical Activity Among Older Adults With and Without Cognitive Impairment in Assisted Living Settings.* J Aging Phys Act, 2021. **29**(3): p. 391-399.

16. van Alphen, H.J., et al., *Older Adults with Dementia Are Sedentary for Most of the Day.* PLoS One, 2016. **11**(3): p. e0152457.

17. Varma, V.R. and A. Watts, *Daily Physical Activity Patterns During the Early Stage of Alzheimer's Disease.* J Alzheimers Dis, 2017. **55**(2): p. 659-667.
