# Supplementary material for: Use of Wearable Technology for Measuring and Characterizing Sedentary Behavior in People With Mild Cognitive Impairment and Dementia: Systematic Review
Source: JMIR Aging. 2026 Jun 25;9:e85361. doi: 10.2196/85361 (PMC13351645; doi:10.2196/85361)
Supplement: Multimedia Appendix 5 [file aging_v9i1e85361_app5.docx]

| 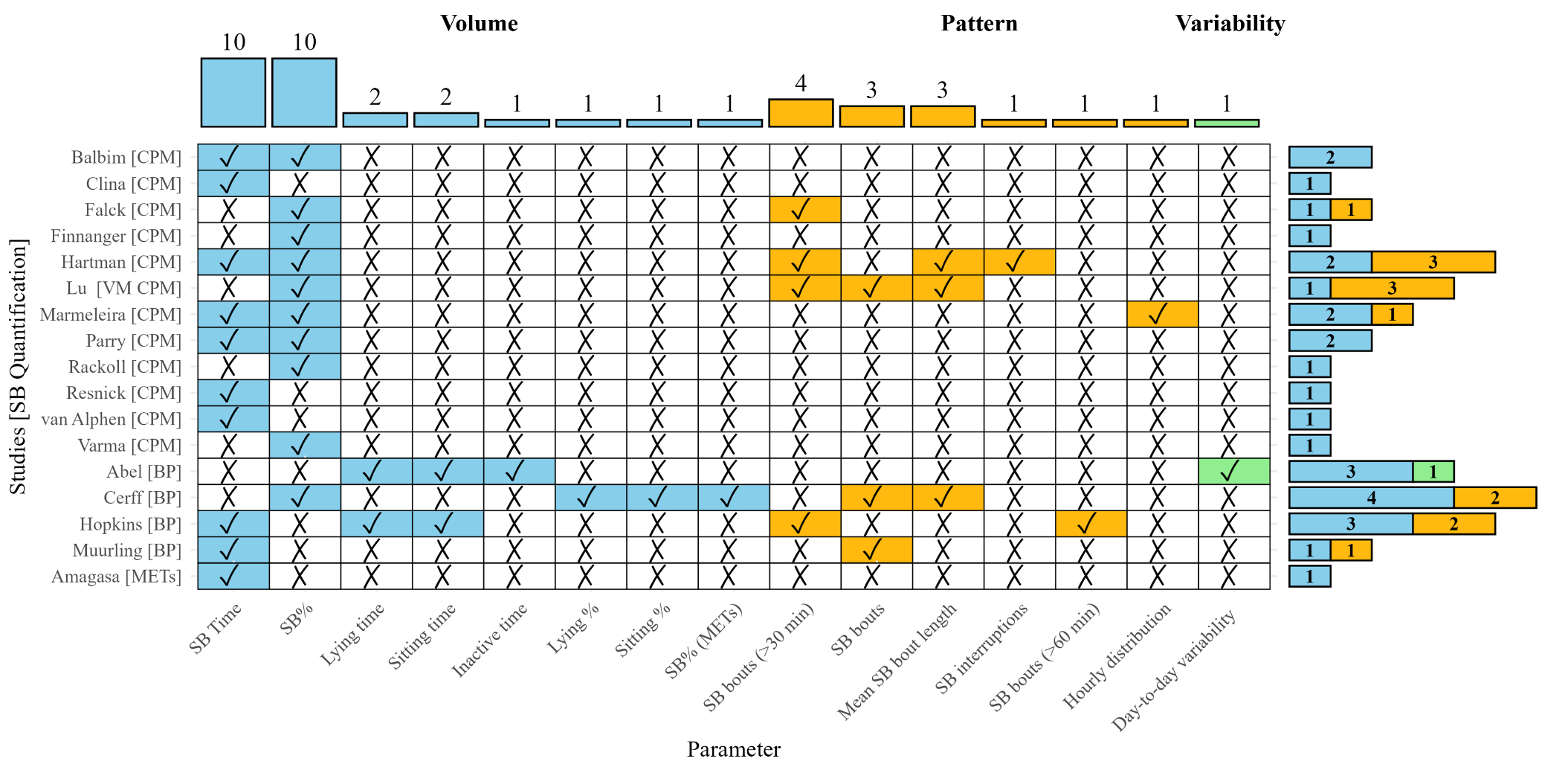 |
| --- |
| **Multimedia Appendix 5:** Matrix of all studies and corresponding parameters used to describe sedentary behaviour.  *Note: studies are sub-grouped based on SB quantification. Top bar chart shows proportion of studies reporting corresponding parameter, right bar chart shows proportion of parameters employed per study, sub-grouped based on categories of SB metrics (volume, pattern, variability). Blue cells and bars correspond to volume parameters, gold bars and cells correspond to pattern parameters and green cells and bars correspond to variability parameters. Abbreviations: CPM: Counts per minute; BP: body posture; METs: Metabolic Equivalent Tasks; %: percentage; min: minutes.* |

References

Abel, B., Pomiersky, R., Werner, C., Lacroix, A., Schäufele, M., & Hauer, K. (2019). Day-to-day variability of multiple sensor-based physical activity parameters in older persons with dementia. *Arch Gerontol Geriatr*, *85*, 103911. <https://doi.org/10.1016/j.archger.2019.103911>

Amagasa, S., Inoue, S., Murayama, H., Fujiwara, T., Kikuchi, H., Fukushima, N., Machida, M., Chastin, S., Owen, N., & Shobugawa, Y. (2020). Associations of Sedentary and Physically-Active Behaviors With Cognitive-Function Decline in Community-Dwelling Older Adults: Compositional Data Analysis From the NEIGE Study. *J Epidemiol*, *30*(11), 503-508. <https://doi.org/10.2188/jea.JE20190141>

Balbim, G. M., Falck, R. S., Boa Sorte Silva, N. C., Kramer, A. F., Voss, M., & Liu-Ambrose, T. (2024). The Association of the 24-Hour Activity Cycle Profiles With Cognition in Older Adults With Mild Cognitive Impairment: A Cross-Sectional Study. *J Gerontol A Biol Sci Med Sci*, *79*(7). <https://doi.org/10.1093/gerona/glae099>

Cerff, B., Maetzler, W., Sulzer, P., Kampmeyer, M., Prinzen, J., Hobert, M. A., Blum, D., van Lummel, R., Del Din, S., Gräber, S., Berg, D., & Liepelt-Scarfone, I. (2017). Home-Based Physical Behavior in Late Stage Parkinson Disease Dementia: Differences between Cognitive Subtypes. *Neurodegener Dis*, *17*(4-5), 135-144. <https://doi.org/10.1159/000460251>

Clina, J. G., Bodde, A. E., Chang, J., Helsel, B. C., Sherman, J. R., Vidoni, E. D., Williams, K. N., Washburn, R. A., Donnelly, J. E., & Ptomey, L. T. (2025). Factors Associated With Physical Activity in Alzheimer’s Disease: A Cross-Sectional Study of Individuals and Their Caregivers. *Journal of Aging and Health*, *0*(0), 08982643251318766. <https://doi.org/10.1177/08982643251318766>

Falck, R. S., Davis, J. C., & Liu-Ambrose, T. (2017). What is the association between sedentary behaviour and cognitive function? A systematic review. *Br J Sports Med*, *51*(10), 800-811. <https://doi.org/10.1136/bjsports-2015-095551>

Finnanger Garshol, B., Ellingsen-Dalskau, L. H., & Pedersen, I. (2020). Physical activity in people with dementia attending farm-based dementia day care – a comparative actigraphy study. *BMC Geriatrics*, *20*(1), 219. <https://doi.org/10.1186/s12877-020-01618-4>

Hartman, Yvonne A. W., Karssemeijer, Esther G. A., van Diepen, Lisanne A. M., Olde Rikkert, Marcel G. M., & Thijssen, Dick H. J. (2018). Dementia Patients Are More Sedentary and Less Physically Active than Age- and Sex-Matched Cognitively Healthy Older Adults. *Dementia and Geriatric Cognitive Disorders*, *46*(1-2), 81-89. <https://doi.org/10.1159/000491995>

Hopkins, J., McVeigh, J., Hill, K., Ellis, K. A., Jacques, A., & Burton, E. (2024). Associations between physical activity, sedentary behaviour and cognitive domain performance of people living with mild cognitive impairment in the community. *Aust Occup Ther J*, *71*(4), 527-539. <https://doi.org/10.1111/1440-1630.12944>

Lu, Z., Harris, T. B., Shiroma, E. J., Leung, J., & Kwok, T. (2018). Patterns of Physical Activity and Sedentary Behavior for Older Adults with Alzheimer's Disease, Mild Cognitive Impairment, and Cognitively Normal in Hong Kong. *J Alzheimers Dis*, *66*(4), 1453-1462. <https://doi.org/10.3233/jad-180805>

Marmeleira, J., Ferreira, S., & Raimundo, A. (2017). Physical activity and physical fitness of nursing home residents with cognitive impairment: A pilot study. *Exp Gerontol*, *100*, 63-69. <https://doi.org/10.1016/j.exger.2017.10.025>

Muurling, M., Badissi, M., de Boer, C., Legdeur, N., Barkhof, F., van Berckel, B. N. M., Maier, A. B., Pijnappels, M., & Visser, P. J. (2023). Physical activity levels in cognitively normal and cognitively impaired oldest-old and the association with dementia risk factors: a pilot study. *BMC Geriatrics*, *23*(1), 129. <https://doi.org/10.1186/s12877-023-03814-4>

Parry, S., Chow, M., Batchelor, F., & Fary, R. E. (2019). Physical activity and sedentary behaviour in a residential aged care facility. *Australas J Ageing*, *38*(1), E12-e18. <https://doi.org/10.1111/ajag.12589>

Rackoll, T., Neumann, K., Passmann, S., Grittner, U., Külzow, N., Ladenbauer, J., & Flöel, A. (2021). Applying time series analyses on continuous accelerometry data-A clinical example in older adults with and without cognitive impairment. *PLoS One*, *16*(5), e0251544. <https://doi.org/10.1371/journal.pone.0251544>

Resnick, B., Boltz, M., Galik, E., Fix, S., & Zhu, S. (2021). Feasibility, Reliability, and Validity of the MotionWatch 8 to Evaluate Physical Activity Among Older Adults With and Without Cognitive Impairment in Assisted Living Settings. *J Aging Phys Act*, *29*(3), 391-399. <https://doi.org/10.1123/japa.2020-0198>

van Alphen, H. J., Volkers, K. M., Blankevoort, C. G., Scherder, E. J., Hortobágyi, T., & van Heuvelen, M. J. (2016). Older Adults with Dementia Are Sedentary for Most of the Day. *PLoS One*, *11*(3), e0152457. <https://doi.org/10.1371/journal.pone.0152457>

Varma, V. R., & Watts, A. (2017). Daily Physical Activity Patterns During the Early Stage of Alzheimer's Disease. *J Alzheimers Dis*, *55*(2), 659-667. <https://doi.org/10.3233/jad-160582>
